# The emergence of a new sex-system (XX/XY_1_Y_2_) suggests a species complex in the “monotypic” rodent *Oecomys auyantepui* (Rodentia, Sigmodontinae)

**DOI:** 10.1038/s41598-022-12706-3

**Published:** 2022-05-24

**Authors:** Willam Oliveira da Silva, Celina Coelho Rosa, Malcolm Andrew Ferguson-Smith, Patricia Caroline Mary O’Brien, Juliane Saldanha, Rogério Vieira Rossi, Julio Cesar Pieczarka, Cleusa Yoshiko Nagamachi

**Affiliations:** 1 Laboratório de Citogenética, Centro de Estudos Avançados da Biodiversidade, Instituto de Ciências Biológicas, Universidade Federal do Pará (UFPA), Belém, Pará Brazil; 2 Department of Veterinary Medicine, Cambridge Resource Centre for Comparative Genomics, University of Cambridge, Cambridge, UK; 3 Departamento de Biologia e Zoologia, Instituto de Biociências, Universidade Federal do Mato Grosso (UFMT), Cuiabá, Mato Grosso Brazil

**Keywords:** Evolution, Evolutionary genetics, Genetics, Cytogenetics, Evolutionary biology

## Abstract

X-autosome translocation (XY_1_Y_2_) has been reported in distinct groups of vertebrates suggesting that the rise of a multiple sex system within a species may act as a reproductive barrier and lead to speciation. The viability of this system has been linked with repetitive sequences located between sex and autosomal portions of the translocation. Herein, we investigate *Oecomys auyantepui*, using chromosome banding and Fluorescence In Situ Hybridization with telomeric and *Hylaeamys megacephalus* whole-chromosome probes, and phylogenetic reconstruction using mtDNA and nuDNA sequences. We describe an amended karyotype for *O. auyantepui* (2n = 64♀65♂/FNa = 84) and report for the first time a multiple sex system (XX/XY_1_Y_2_) in Oryzomyini rodents. Molecular data recovered *O. auyantepui* as a monophyletic taxon with high support and cytogenetic data indicate that *O. auyantepui* may exist in two lineages recognized by distinct sex systems. The Neo-X exhibits repetitive sequences located between sex and autosomal portions, which would act as a boundary between these two segments. The G-banding comparisons of the Neo-X chromosomes of other Sigmodontinae taxa revealed a similar banding pattern, suggesting that the autosomal segment in the Neo-X can be shared among the Sigmodontinae lineages with a XY_1_Y_2_ sex system.

## Introduction

Chromosomal rearrangements are drivers in karyotypic evolution and are often associated with speciation^1–5^. Mammals are known to exhibit a stable sex determination system, but distinct sex-autosome translocations may have triggered the separation of Theria and Prototheria (monotremes) (190 MYA) and between Eutheria (placental mammals) and Metatheria (marsupials) (166 MYA)^6^. Although the euchromatic region of the X chromosome is considered conserved among highly rearranged karyotypes of placental mammals^6^, recent investigations on Arvicolinae (Myomorpha) rodents have shown that the X chromosome has undergone several intrachromosomal rearrangements, such as centromere shifts, peri- and paracentric inversions, that were also accompanied by repetitive sequences^7^. Regardless of whether chromosomal rearrangements are the primary cause of speciation^2,8^, or whether karyotypic divergence between closely related species are a casualty of the speciation process^9,10^, the most deleterious among the speciation-linked rearrangements^11,12^ are tandem translocations, reciprocal translocations^13,14^ and X-autosome translocations^15,16^.

The rise of an X-autosome translocation is subordinated to the same epigenetic mechanism that guarantees dosage compensation between normal females (XX) and males (XY) by silencing one of the Xs in females^17^. In this type of event, the inactivation progress in one of the X chromosomes of females^18^ spreads to the autosomal segment translocated to the X, silencing genes in the autosomal portion^19^ generating deletion/duplications with deleterious effects^20^.

Although deleterious effects of sex-autosome translocations have been described in the literature for humans and mice (e.g., male sterility; embryonic lethality)^15,21,22^, this type of chromosomal rearrangement has been reported in natural populations of distinct groups of vertebrates, such as fish^23,24^, anurans^25^ and mammals^17,26^. The presence of intercalary heterochromatic blocks between autosomal and ancestral X chromosome segments could suppress the X-inactivation progress in the autosomal segment, allowing viability in this system^16,27–29^. Several studies have shown the presence of heterochromatic blocks, telomeric repeats and/or rDNA (ribosomal DNA) clusters in different mammalian lineages that exhibit X-autosome translocation, for example in bats (Chiroptera) of genera *Artibeus*, *Carollia*, and *Uroderma*^29–32^; in rodents (Rodentia) of genera *Nannomys* (Muridae)^17^, *Proechimys* (Echimyidae)^33–35^, and *Taterillus* (Muridae)^16^; in diprotodont marsupials (Diprotodontia) of genus *Wallabia*^36^; and in ruminants (Artiodactyla) of genera *Antilope* and *Gazella*^37^.

In rodents from the Brazilian Amazon, the XX/XY_1_Y_2_ multiple sex system has been reported only in two genera from the Echimyidae family: *Lonchothrix*^38^ and *Proechimys*^33–35^. In *Lonchothrix emiliae*, the multiple sex system was identified based on classic banding^38^, while in the *Proechimys* taxa it was detected by FISH (Fluorescence In Situ Hybridization) with whole chromosome probes (chromosome painting) from *P. roberti* and *P. goeldii*^35^. In Sigmodontinae rodents (Rodentia, Cricetidae), the Oryzomyini tribe currently comprises 29 genera and is the most diverse of the 11 tribes within the subfamily^39–41^, but multiple sex systems are acknowledged solely in representatives of the Akodontini, Phyllotini and Reithrodontini tribes: *Deltamys kempi* (Akodontini) exhibits a X_1_X_1_X_2_X_2_/X_1_X_2_Y sex system due to a translocation involving chromosomes 2 and Y^42^; *Salinomys delicatus* (Phyllotini) shows a XY_1_Y_2_ system^43^; and *Reithrodon* (Reithrodontini) exhibits a XY_1_Y_2_ system (Uruguay population) and a Neo-XY system (Brazil population)^44^.

In Oryzomyini, the genus *Oecomys* has been particularly challenging in taxonomy, distribution patterns and speciation mechanisms. Comprising 19 species to date, *Oecomys* has been investigated using several approaches, such as morphology, nuclear DNA (nuDNA), mitochondrial DNA (mtDNA), and cytogenetics, which have shown that some lineages correspond to species complexes^39,45–51^. *Oecomys auyantepui* has been recognized as a monophyletic lineage and a monotypic taxon^48,52^. The species is distributed from southeastern Venezuela to north-central Brazil, in the Guiana subregion of Amazonia^39^, and exhibits two sympatric populations with distinct diploid numbers (2n) of 64 and 66 and autosomal fundamental numbers (FNa) of 110 and 114, respectively^52^. A third karyotype of 2n = 72/FNa = 80 was described^53^. In addition, an interstitial telomeric sequence (ITS) was identified at the centromeric region of the bi-armed X chromosomes in karyotypes with 2n = 64 and 66, which suggests that chromosomal rearrangements have driven the evolution of this chromosome in *O. auyantepui*^52^.

It is noteworthy that cytogenetics studies with *Oecomys* have shown a substantial diversity in 2n and FNa, ranging from 54 to 86 and from 62 to 140, respectively^39,45,47,48,50,52–55^. However, specific events that shaped extant karyotypes remain unclear for most species, except for *O. catherinae* from Pará (OCA-PA; 2n = 62/FNa = 62), *O. catherinae* from Rio de Janeiro (OCA-RJ; 2n = 60/FNa = 62), *O. paricola* cytotype A (OPA-A; 2n = 72/FNa = 75), *O. paricola* cytotype B (OPA-B; 2n = 70/FNa = 75), and *O. paricola* cytotype C (OPA-C; 2n = 70/FNa = 72) that were investigated by chromosome painting with *Hylaeamys megacephalus* whole chromosome probes (HME; Oryzomyini)^47,50^. In addition to elucidating the chromosomal rearrangements that occurred in these species, the chromosome painting analysis helped to delineate taxonomic limits, as the authors^47,50^ were able to identify a hidden diversity and proposed that *O. catherinae* and *O. paricola* “eastern clade” were composed of two and three species, respectively.

Considering the evolutionary force of chromosomal rearrangements regarding speciation and diversification of species, we set out to investigate if the emergence of a new sex-system triggered the speciation process in the monotypic taxon *Oecomys auyantepui*.

In order to achieve this goal, we used classic cytogenetics, telomeric and HME whole chromosome probes^54^, mtDNA (mitochondrial DNA) and nuDNA (nuclear DNA) sequences. Here we discuss the chromosomal evolution of the genus, and report for the first time a multiple sex system (XX/XY_1_Y_2_) in Oryzomyini rodents. We also compared the taxa from the present study with other species analyzed elsewhere using the same set of probes^47,50,54–59^.

## Results

### Classic and molecular cytogenetics

**Figure 1:**
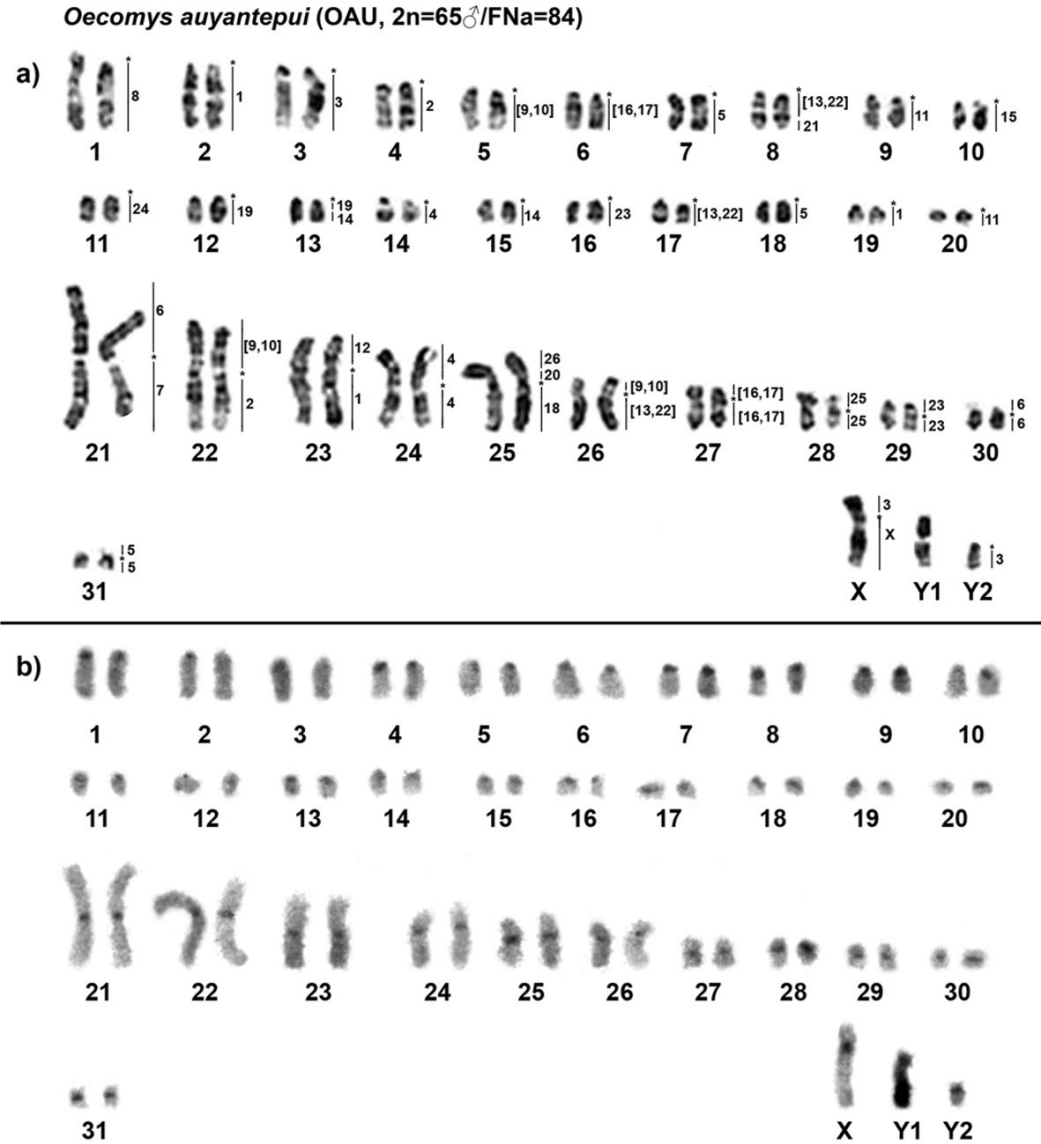
*Oecomys auyantepui* (2n = 65♂/FNa = 84) (**a**) G-banded karyotype with chromosome painting revealed by *Hylaeamys megacephalus* (HME) whole chromosome probes^54^, and (**b**) C-banded karyotype. An asterisk indicates a centromere.

*Oecomys auyantepui* (OAU) has a 2n = 64♀65♂/FNa = 84 karyotype, with a multiple sex system (XX/XY_1_Y_2_). The autosomal set consists of 20 acrocentric pairs (1–20) and 11 meta/submetacentric pairs (21–31). In females sex chromosomes were recognized as two medium-sized submetacentric Neo-X chromosomes; in males sex chromosomes were identified as one Neo-X and two Ys: Y_1_ chromosome was a medium submetacentric (original Y) and Y_2_ was a small acrocentric (Xp homologue) (Fig. 1a). The constitutive heterochromatin (CH) is distributed in the centromeric regions of almost all autosomes, the Neo-X and Y_2_ chromosomes. The CH is a small region in most of the autosomes and the Y_1_ chromosome has a large heterochromatic block in the long arm (Fig. 1b).

Cross-species FISH with HME probes yielded 42 signals on the OAU chromosomes (Fig. 1a, Table 1, see Supplementary Figs. 1 and 2). Ten autosomal probes are conserved; of them, four (HME 8, 15, 24 and 25) hybridize to whole chromosomes of OAU (1, 10, 11 and 28, respectively) and six (HME 7, 12, 18, 20, 21 and 26) hybridized with portions of chromosomes of OAU (21q, 23p, 25q, 25p proximal, 8q distal and 25p distal, respectively). Twelve autosomal probes show multiple signals in OAU: HME 1 hybridize to OAU 2, 19 and 23q; HME 2 hybridize to OAU 4 and 22q; HME 4 hybridize to OAU 14 and 24; HME 5 hybridize to OAU 7, 18 and 31; HME 6 hybridize to OAU 21p and 30; HME (9,10) hybridize to OAU 5, 22p and 26p; HME 11 hybridize to OAU 9 and 20; HME (13,22) hybridize to OAU 8q proximal, 17 and 26q; HME 14 hybridize to OAU 13q distal and 15; HME (16,17) hybridize to OAU 6 and 27; HME 19 hybridize to OAU 12 and 13q proximal; HME 23 hybridize to OAU 16 and 29.

**Table 1:** FISH results for *Oecomys auyantepui* (OAU; 2n = 65♂/FNa = 84), as assessed based on hybridization with *Hylaeamys megacephalus* (HME) whole-chromosome probes^54^.

HME	OAU
1	2, 19, 23q
2	4, 22q
3	3, Xp, Y_2_
4	14, 24
5	7, 18, 31
6	21p, 30
7	21q
8	1
(9,10)	5, 22p, 26p
11	9, 20
12	23p
(13,22)	8q prox., 17, 26q
14	13q dist., 15
15	10
(16,17)	6, 27
18	25q
19	12, 13q prox
20	25p prox
21	8q dist
23	16, 29
24	11
25	28
26	25p dist
X	Xq

The HME 3 probe hybridizes to the short arm of the Neo-X chromosome (OAU Xp), and also hybridizes to OAU 3, and Y_2_; the HME X chromosome hybridizes to the long arm of the Neo-X (OAU Xq).

**Figure 2:**
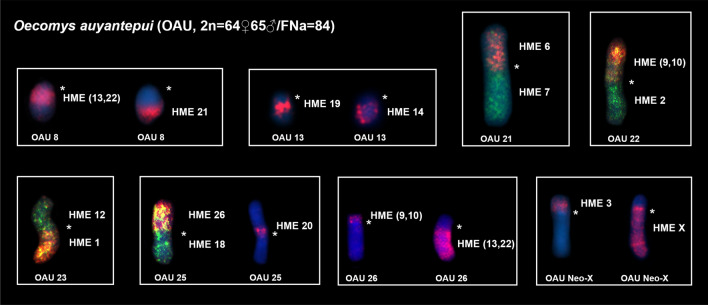
FISH results obtained from *O. auyantepui* (OAU; 2n = 64♀65♂/FNa = 84), using HME whole chromosome probes^54^. Each box corresponds to an OAU pair shown in Fig. 1a that corresponded to more than one HME homologue. Single or multiple images are presented to exhibit full coverage with HME probes on OAU chromosomes. OAU chromosomal pairs identification are shown below the chromosomes, while HME probes are shown beside the chromosomes. An asterisk indicates a centromere. HME whole chromosome probes are shown in green (FITC) and red (CY3); the counterstaining is blue (DAPI).

Seven OAU autosomal pairs show hybridization signals to multiple HME probes: OAU 8 (HME (13,22)/21); OAU 13 (HME 19/14); OAU 21 (HME 6/7); OAU 22 (HME (9,10)/2); OAU 23 (HME 12/1); OAU 25 (HME 26/20/18); OAU 26 (HME (9,10)/(13,22)) (Fig. 2).

**Figure 3:**
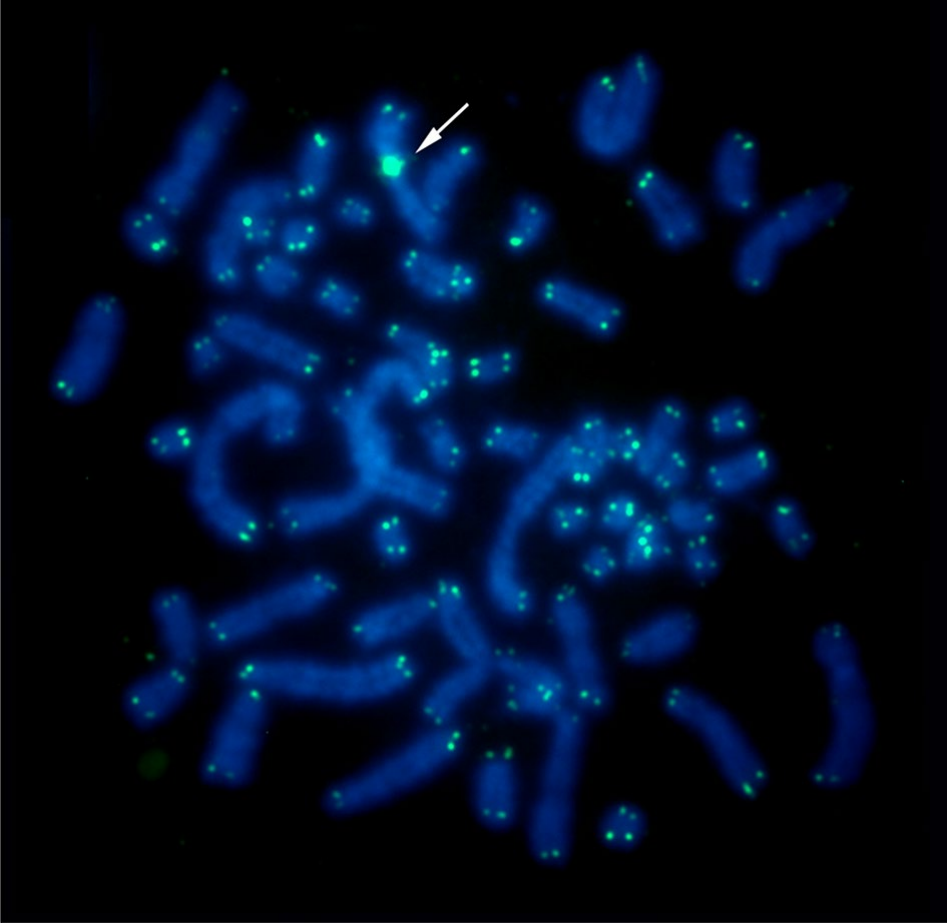
FISH results obtained from *O. auyantepui* (OAU; 2n = 65♂/FNa = 84), using telomeric probes. Arrow indicates the interstitial telomeric sequence (ITS) at the Neo-X chromosome. Telomeric probes are shown in green (FITC); the counterstaining is blue (DAPI).

FISH with telomeric probes showed hybridization signals at the distal regions of all chromosomes, plus a large interstitial telomeric sequence (ITS) at the centromere of the Neo-X chromosome (Fig. 3).

### Phylogenetic analysis

**Figure 4:**
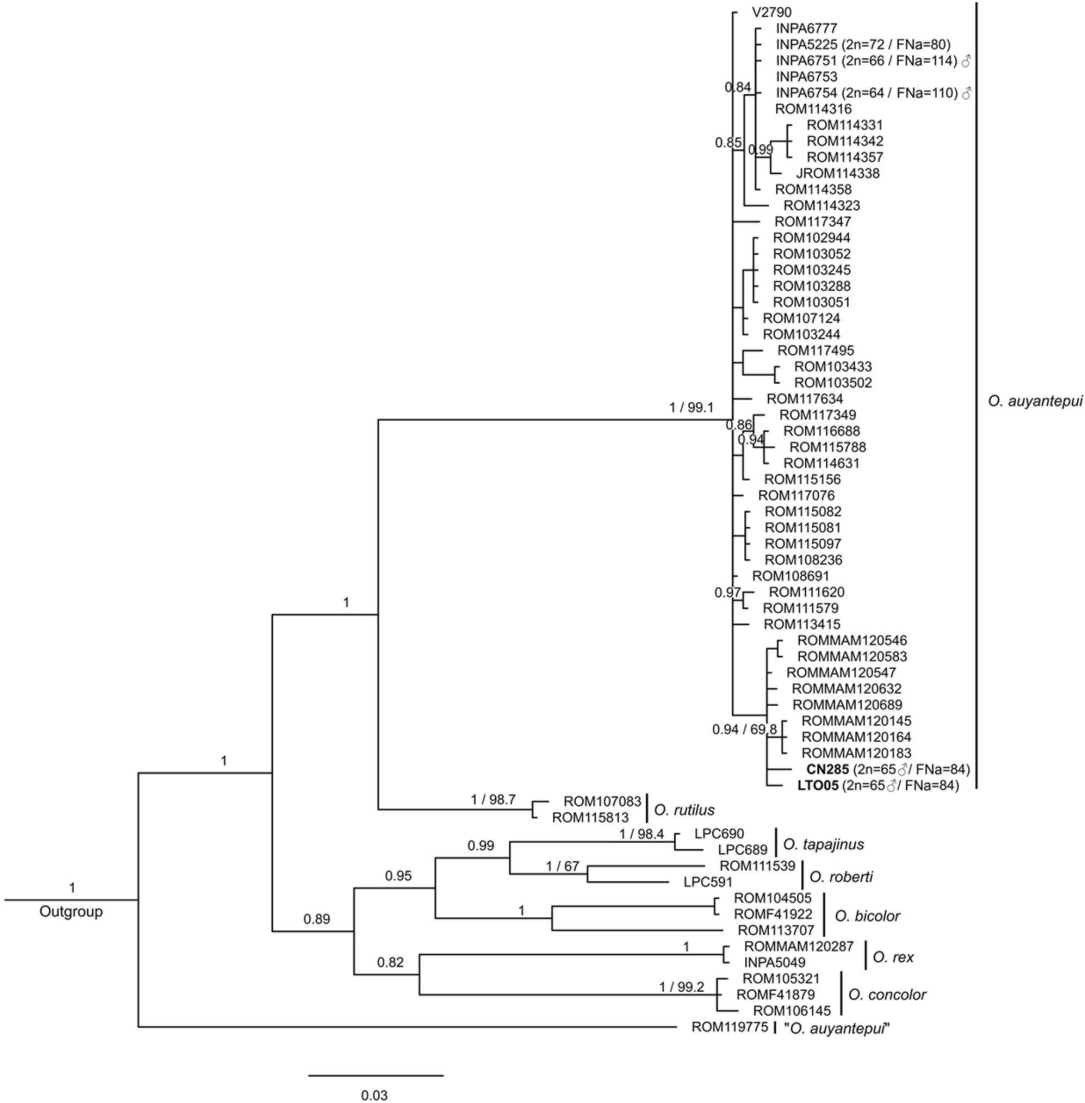
Bayesian Inference topology based on Cytochrome C Oxidase Subunit I. The numbers above branches indicate posterior probability values for Bayesian Inference analysis (only values > 0.80 are shown) and bootstrap values for Maximum Likelihood analysis (only values > 65% are shown). Bold numbers indicate the samples from this study. Sample data are provided in Supplementary Table 1.

**Figure 5:**
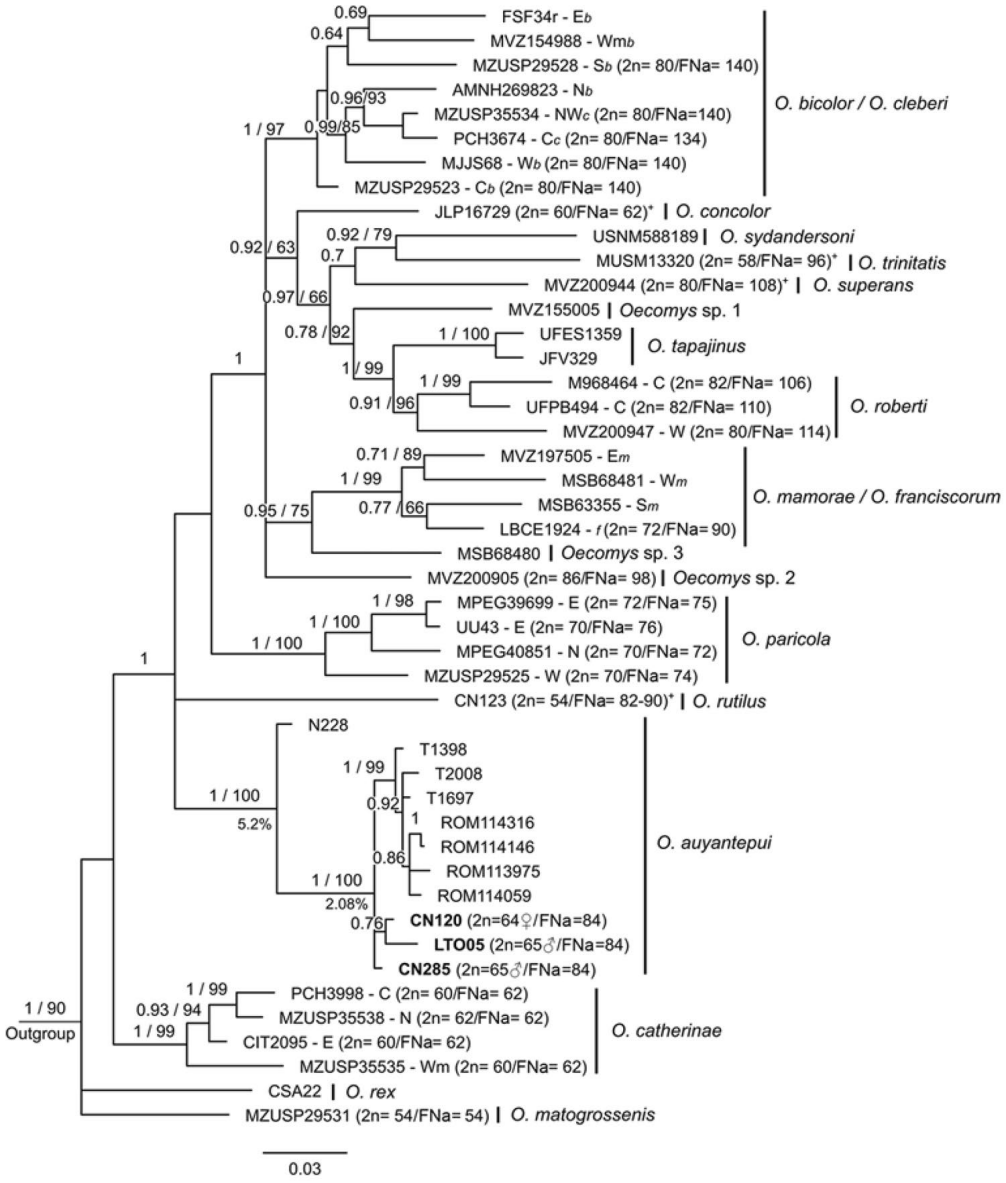
Bayesian Inference topology based on Cytochrome b. The numbers above branches indicate posterior probability values for Bayesian Inference analysis (only values > 0.60 are shown) and bootstrap values for Maximum Likelihood analysis (only values > 60% are shown). Bold numbers indicate the samples from this study. Percentage values are mean genetic distances (p-distances) between high supported selected lineages of *O. auyantepui*. Representatives of different lineages within *O. bicolor*, *O. cleberi*, *O. roberti*, *O. mamorae*, *O. paricola*, and *O. catherinae* complexes are indicated by the letters C (central clade), E (eastern clade), N (northern clade), NW (northwestern clade), S (southern clade), W (western clade), and Wm (westernmost clade), following^48^. Subscribed letters *b* and *c* indicate lineages attributed by^48^ to *O. bicolor* and *O. cleberi*, respectively. Subscribed letters *f* and *m* indicate lineages attributed by^48^ to *O. franciscorum* and *O. mamorae*, respectively. Cross (^+^) denotes karyotype information not obtained from the specimen included in the phylogenetic analysis. Sample data are provided in Supplementary Table 1.

**Figure 6:**
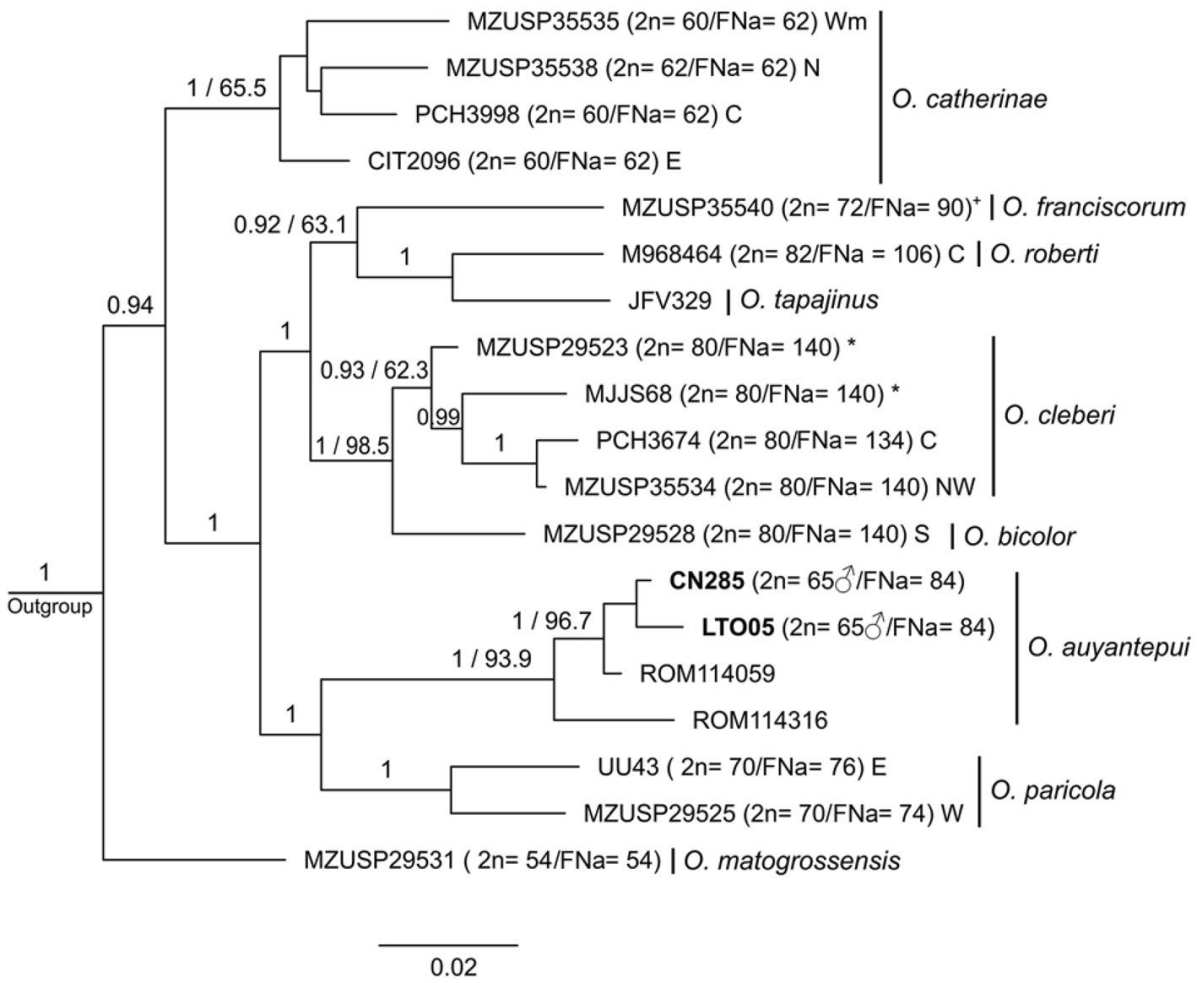
Bayesian Inference topology based on mitochondrial Cytochrome b and nuclear beta-fibrinogen intron 7 concatenated. The numbers above branches indicate posterior probability values for Bayesian Inference analysis (only values > 0.90 are shown) and bootstrap values for Maximum Likelihood analysis (only values > 60% are shown). Bold numbers indicate the samples from this study. Representatives of different lineages within *O. bicolor*, *O. cleberi*, *O. roberti*, *O. paricola*, and *O. catherinae* complexes are indicated by the letters C (central clade), E (eastern clade), N (northern clade), NW (northwestern clade), S (southern clade), W (western clade), and Wm (westernmost clade), following^48^. Asterisk (*) denotes specimens identified as *O. bicolor* by^48^. Cross (^+^) denotes karyotype information not obtained from the specimen included in the phylogenetic analysis. Sample data are provided in Supplementary Table 1.

A more detailed phylogenetic analysis of the genus *Oecomys* was already proposed^48^. Thus, in this work we focused on *O. auyantepui* and representatives of each *Oecomys* species/clade recognized in the literature^48,49,51^ (Supplementary Table 1). The genus *Oecomys* was recovered as monophyletic in the topologies obtained with the Cytochrome b (Cytb) dataset, the Cytochrome C Oxidase Subunit I (COI) dataset, and with the concatenated dataset (Cytb + beta-​fibrinogen intron 7 [FGB-I7]), with high support values recorded only in the Bayesian Inference (BI) analyses (Figs. 4, 5, 6). In the Cytb topology, lineages of *O. bicolor* and *O. cleberi* were not recovered as reciprocally monophyletic, as well as lineages of *O. mamorae* and *O. franciscorum* (Fig. 5).

We found mean interspecific p-distances ranging from 6.47% (between *O. concolor* and *Oecomys* sp.2) to 14.80% (between *O. franciscorum/O. mamorae* and *O. matogrossensis*), and mean intraspecific p-distances varying from zero in several species to 6.2% in *O. bicolor*/*O. cleberi* (2.15% in *O. auyantepui*, particularly; Table 2).

**Table 2:** Mean genetic p-distances between sequences of *Oecomys* based on the mitochondrial gene Cytochrome b.

		1	2	3	4	5	6	7	8	9	10	11	12	13	14	15	16	17
1	*O. auyantepui*	**2.15**	2.06	2.08	2.36	2.10	2.31	1.87	2.25	2.14	2.36	2.31	2.24	2.32	2.43	2.23	2.29	2.26
2	*O. bicolor /O. cleberi*	13.60	**6.20**	1.58	1.35	1.69	2.02	1.75	2.08	1.59	1.84	1.62	1.90	1.68	1.96	1.43	1.62	1.67
3	*O. catherinae*	11.95	9.75	**4.39**	1.73	1.77	1.73	1.69	1.73	1.99	2.01	2.13	1.97	2.20	2.19	2.03	1.89	1.96
4	*O. concolor*	14.16	6.90	8.58	–	1.58	2.41	1.88	2.30	1.95	1.99	2.07	1.95	2.09	1.95	1.92	1.75	1.80
5	*O. franciscorum / O. mamorae*	12.57	10.84	10.54	8.33	**5.97**	2.21	1.89	2.15	1.89	2.06	2.00	1.73	1.89	1.75	1.80	1.73	2.02
6	*O. matogrossensis*	13.98	12.81	8.83	13.43	14.80	–	1.94	2.18	2.19	2.28	2.24	2.37	2.26	2.28	2.41	2.42	2.47
7	*O. paricola*	10.67	11.21	9.20	9.45	10.95	11.32	**3.73**	1.91	1.86	2.20	2.00	2.01	2.20	1.99	2.03	1.95	2.00
8	*O. rex*	13.43	12.75	8.46	12.44	12.81	11.44	10.45	–	2.21	2.31	2.26	2.28	2.48	2.28	2.22	2.33	2.20
9	*O. roberti*	12.75	9.56	11.36	9.78	11.24	12.94	10.45	12.77	**3.65**	2.14	1.78	2.01	1.55	2.14	1.77	1.98	2.20
10	*O. rutilus*	14.07	10.57	10.82	8.96	12.81	11.44	12.94	12.94	12.11	–	2.14	2.14	2.10	2.36	2.21	2.09	2.02
11	*O. superans*	14.20	8.71	11.57	9.45	11.69	11.94	10.57	11.94	8.29	10.45	–	2.37	2.18	2.22	2.00	2.14	2.15
12	*O. sydandersoni*	12.75	11.07	10.95	8.96	9.45	13.43	10.95	12.44	10.45	10.95	12.44	–	2.08	1.89	2.04	1.90	2.13
13	*O. tapajinus*	13.23	9.39	12.69	9.70	10.70	12.69	12.69	14.18	6.72	10.20	11.19	10.20	**0.50**	2.13	1.77	2.10	2.31
14	*O. trinitatis*	14.65	12.00	13.31	8.96	9.58	12.44	10.32	12.44	11.77	12.94	11.94	8.46	10.70	–	2.26	2.03	2.20
15	*Oecomys* sp.1	12.98	7.59	11.69	8.96	10.20	14.43	11.82	11.94	8.46	11.94	9.45	9.45	6.97	11.94	–	2.12	2.26
16	*Oecomys* sp.2	13.34	9.08	9.70	6.47	9.08	14.43	10.07	12.44	9.78	9.95	10.45	8.46	10.20	9.45	10.45	–	1.94
17	*Oecomys* sp.3	12.75	8.40	10.32	6.97	11.44	14.43	10.45	11.44	12.27	9.45	9.95	10.45	11.69	11.44	11.44	8.46	–
	Outgroup	17.26	14.57	14.05	13.43	16.46	14.10	15.05	12.60	14.93	15.26	15.26	15.42	15.09	15.59	13.76	15.26	16.09

Intraspecific distances are in bold, and standard deviation values are above diagonal. Values in percentage.

In all three datasets, *O. auyantepui* was recovered as a monophyletic taxon with high support. The COI phylogeny was the only one that included all *O. auyantepui* karyotyped samples from this work and from the literature^51,52^, as well as most sequences available on GenBank (Supplementary Table 1). In the COI topology, specimens of *O. auyantepui* formed a polytomy, with no resolution among most specimens, including those with similar karyotypes (Fig. 4). In the Cytb topology, the specimen N228 was recovered as the most divergent within *O. auyantepui*, with 5.20% of mean genetic divergence from its conspecifics. The remaining specimens formed a subclade with neither resolution nor support among most of the specimens. As in the COI topology, specimens with similar karyotypes did not nest in a subclade (Fig. 5). Finally, in the concatenated data topology, the specimen ROM 114,316 was the first one to diverge, and the specimen ROM 114,059 was recovered as sister to the 2n = 65♂/FNa = 84 specimens included in this analysis. Although these latter specimens appeared as a subclade, there was no support for that (Fig. 6).

## Discussion

### Chromosomal evolution and signatures in Oecomys (Rodentia, Sigmodontinae)

As mentioned above, there is a large variation in 2n from 54 to 86 and in FNa from 62 to 140 among the *Oecomys* species, with karyotypes mainly composed of one-armed chromosomes^45,47,48,50,52,53,60,61^. The variation in 2n and FNa occur both within and between species, indicating that fusions/fissions, pericentric inversions (or centromeric repositioning), translocations, and addition/deletion of constitutive heterochromatin are the main forces acting in the chromosomal evolution of this group of Sigmodontinae rodents. Thus, we used chromosome painting to make a comparative analysis of taxa in the present study (*O. auyantepui*) and in the literature (*O. catherinae* and *O. paricola*) to precisely identify the rearrangements among them^47,50^.

As a result, chromosome painting analysis in the karyotypes of *O. auyantepui* (OAU), *O. paricola* (OPA-A, OPA-B and OPA-C) and *O. catherinae* (OCA-PA and OCA-RJ) (Supplementary Table 2) showed that the chromosomal variation in 2n from 60 to 72 and in FNa from 62 to 84 are due to 23 fusion/fission events, four translocations, seven pericentric inversions and amplification/deletion of constitutive heterochromatin on two autosomal syntenic blocks plus the X chromosome (Supplementary Fig. 3), with only seven syntenic blocks conserved without detectable rearrangements. Remarkably, we observed that the rearrangements that differentiate OPA cytotypes (OPA-A, OPA-B, and OPA-C) from each other are different from those responsible for the variability between OCA cytotypes (OCA-PA and OCA-RJ) and OAU. This suggests that the rearrangements mainly occurred in distinct syntenic blocks among these species (Supplementary Fig. 3). Consequently, we propose that each of these three species has evolved independently and has not followed the same path of rearrangements or the same chromosomes.

Moreover, by detecting an elevated number of chromosomal rearrangements among three taxa (*O. auyantepui*, *O. catherinae*, and *O. paricola*) with not-so-distant 2n (from 60 to 72), we assume that the chromosomal evolution in *Oecomys* is more complex than previously thought. In this sense, the use of HME whole chromosome probes in representatives of the main lineages of *Oecomys* provides a more accurate view of chromosomal evolution, and associated with detailed phylogeographic studies, is a key factor in understanding the speciation processes of this diverse and speciose group of Sigmodontinae rodents.

By comparing the OAU karyotype with the other Sigmodontinae species investigated by HME whole chromosome probes (Supplementary Table 2), we identified that OAU exhibited the chromosomal signatures proposed for the *Oecomys* genus, namely the fragmentation of HME 1 into three blocks and the syntenic block HME (13,22)/21^50^. We also detected an exclusive trait for OAU, the syntenic block HME 26/20/18, which is different from those described for OCA (HME (9,10)/14/5, 23/19/11 and 26/11) and OPA (HME 4/19); OAU also shared and the fragmentation of HME 3 into two blocks with OPA, previously described as exclusive for this species^50^. Among the eleven chromosomal signatures proposed for the Sigmodontinae subfamily (HME 7/(9,10), 8, 1/12, 6/21, 11/(16,17), 5/(16,17), 20/(13,22), 15, 19/14/19, 24, and 26)^47,50,61,69–73^, OAU exhibits only five: HME 8, 15, 24 and 1/12, plus the chromosomal signature HME 19/14/19, which is present as a derived character due to a fission that generates the HME 19/14 (OAU 13) and 19 (OAU 12).

### New sex system in Oecomys auyantepui (XX/XY_1_Y_2_)

In the genus *Oecomys*, sex chromosomes variation in size and morphology are frequently related to the addition/deletion of CH, which is common in rodents^62^; the one-armed X chromosome often exhibits CH at the centromeric region, while the bi-armed form presents a heterochromatic block in the short arm and the variation in length of the Y is often due to the size of the heterochromatic block in the long arm^45,47,48,50,52^. However, the bi-armed X chromosome of *O. auyantepui* from the present study exhibits an euchromatic short arm, with CH at the centromere (Fig. 1b). This indicates that events other than the general addition/deletion of CH were involved in this variation, and this is supported by the HME 3 hybridization signal in the short arm of OAU Neo-X (Xp) and Y_2_ (see Supplementary Fig. 1), validating the emergence of a new sex system (XX/XY_1_Y_2_) in *O. auyantepui* (2n = 64♀65♂/FNa = 84). Regarding Sigmodontinae rodents, this is the first record of a multiple sex system in Oryzomyini, since this type of sex determination system has previously been reported only in representatives of Akodontini (X_1_X_1_X_2_X_2_/X_1_X_2_Y)^42^, Phyllotini (XY_1_Y_2_)^43^ and Reithrodontini (XY_1_Y_2_ and Neo-XY)^44^ tribes.

**Figure 7:**
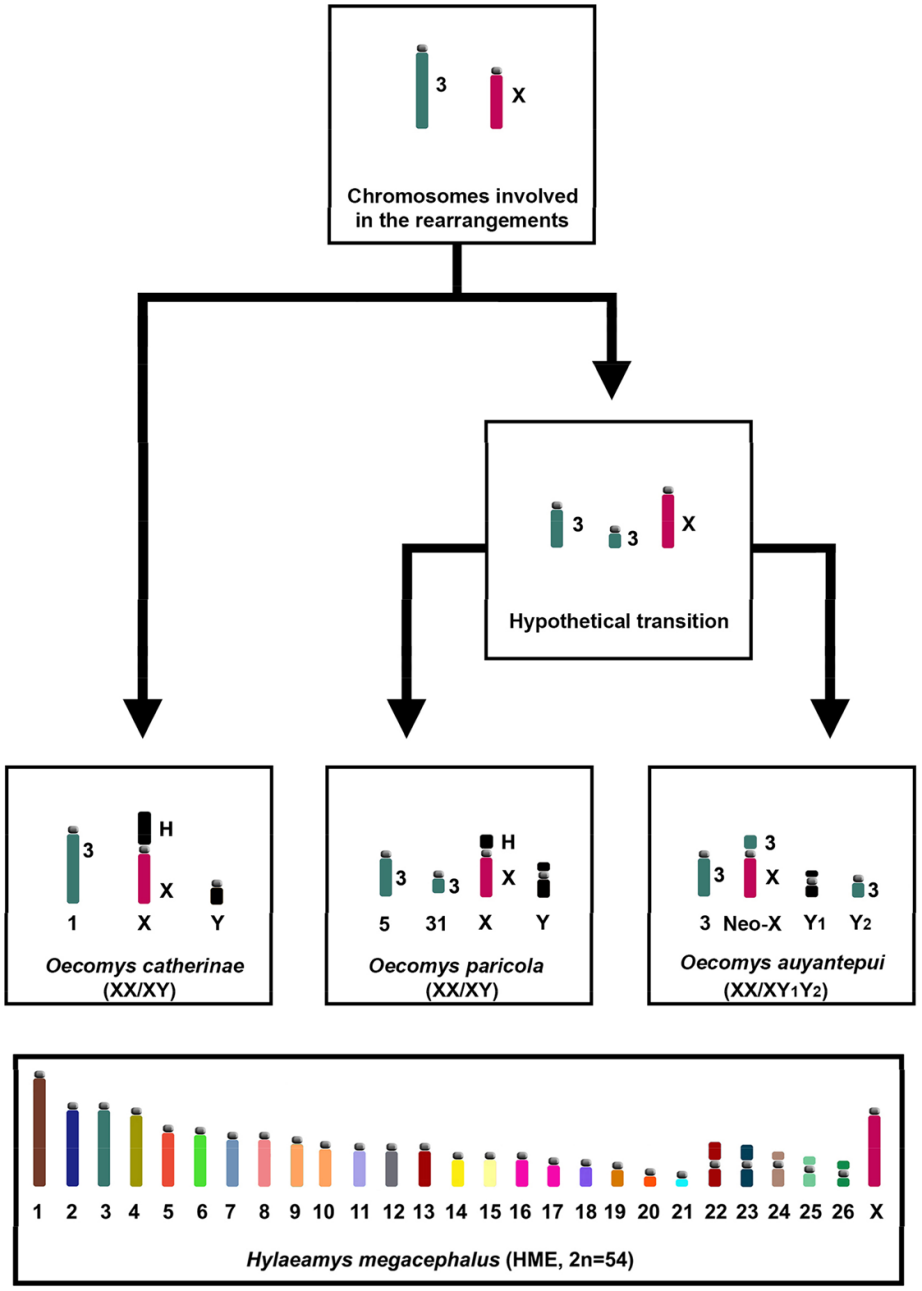
Idiograms of the chromosomes involved in the emergence of a new sex-system (XX/XY_1_Y_2_) in *Oecomys auyantepui* (OAU; 2n = 64♀65♂/FNa = 84), as assessed based on HME probes^54^. Idiograms in black (Y and Y_1_) were evaluated only by classical cytogenetics. Arrows show direction of chromosome change. We used *O. paricola*-cytotype A^50^ and *O. catherinae*-Pará^47^ as representatives of their respective species. Numbers below idiograms correspond to the identification of the chromosomal pair; numbers beside idiograms correspond to the HME probes. The bottom box encompasses an idiogram of HME karyotype previously elaborated^58^ and adapted in the present study. Each HME chromosome is shown with a single color, except the pairs (9,10), (13,22) and (16,17), which have one color each. (H) Indicates large block of constitutive heterochromatin.

In order to understand the Neo-X origin in *O. auyantepui*, we constructed a dendrogram that shows a hypothetical scenario with the chromosomes involved. We made a comparative analysis among the other Sigmodontinae taxa studied with the same set of probes (Supplementary Table 2) and considered as outgroup the 16 karyotypes from the genera *Akodon*, *Blarinomys*, *Necromys, Oxymycterus*, *Thaptomys* (Akodontini), *Cerradomys*, *Hylaeamys*, and *Neacomys* (Oryzomyini), while as ingroup we considered the karyotypes of *Oecomys catherinae*, *O. paricola* and *O. auyantepui* (Oryzomyini). Except for *Cerradomys*, all karyotypes from the outgroup showed a HME 3 hybridization signal in one large acrocentric chromosome pair. The X was acrocentric, or bi-armed with a heterochromatic block in the short arm. Thus, we propose that: (1) the ancestral forms of the HME 3 and X chromosomes were medium acrocentrics; (2) *O. catherinae* maintained the autosomal ancestral form, while the X exhibited an addition of CH in the short arm; (3) the HME 3 divided by fission into two blocks of unequal size (one large and one small) before the diversification events that led to the *O. paricola* and *O. auyantepui* species; (4) in *O. paricola*, the HME 3 small block remained as an independent chromosome, and the X exhibited an addition of CH in the short arm; (5) in *O. auyantepui*, a Robertsonian translocation between the HME 3 small block with the acrocentric X formed the Neo-X chromosome (Fig. 7).

The proposal that intercalary heterochromatic blocks, telomeric repeats and/or rDNA clusters between the ancestral X and the translocated autosome served as a boundary that suppresses the X-inactivation process in the autosomal portion^16,17,28–30^ is in accordance with our results, which show a centromeric heterochromatic block (Fig. 1b) and a large block of ITS in the Neo-X chromosome of *O. auyantepui* (Fig. 3). In the rodent *Mus minutoides* (Muridae) immunofluorescence techniques demonstrated patterns of histone modification in three types of sex chromosomes (Y, X and a mutant X) that confirmed that the X-inactivation does not spread into the translocated autosomal portion^17^. This feature is of prime importance and guarantees the viability of this multiple sex system. In fact, several studies have described natural populations of vertebrates (e.g., bats, rodents, marsupials and ruminants) that possess this type of rearrangement^16,17,26,30–32,35–37,63^; in cases where such repetitive sequences are absent, deleterious effects such as poor viability and infertility are observed^15,16^. The contribution of repetitive sequences in the evolution of the X chromosome was also proposed^7^. The authors microdissected the *Terricola savii* X chromosome into five specific-region probes, hybridized in 20 species of Arvicolinae rodents (Myomorpha), and identified multiple intrachromosomal rearrangements, such as centromere shifts, peri- and paracentric inversions, which were related to the amplification and distribution of repetitive sequences among the Xs of distinct Arvicolinae species^7^.

Exceptions from this proposal are documented in the common ancestor of eutherian mammals, since a sex-autosome translocation occurred and it may have triggered the separation between Eutheria (placental mammals) and Metatheria (marsupials) (166 MYA)^6^, with no intercalary heterochromatic block found between the X and autosomal ancestral segments. Distinct processes from the intercalary heterochromatic block would be involved in the regulation of X-autosome viability and in the suppression of deleterious effects^16^.

It is noteworthy that telomeric repeats occur at the ends of chromosomes where they provide stability, while ITS are relics of chromosomal fusions that arose during karyotype evolution^64^. Thus, although we have identified other chromosomes resulting from Robertsonian translocations in *O. auyantepui* (OAU 21–23, 25; Figs. 2 and 3), the ITS was found only in the Neo-X. An investigation of the effects of these telomeric sequences on gene expression, recombination and rearrangements, was made by introducing 800 bp of the telomere repeat (TTAGGG) in the adenosine phosphoribosyltransferase (APRT) gene in Chinese hamster ovary cells^65^. The main result was that gene rearrangements were greatly increased^65^. This type of chromosome instability is in accordance with the proposal that het-ITS (heterochromatic-ITS) seem to be intrinsically prone to breakage^66^, and that ITSs are hotspots for chromosomal rearrangements^64^. Therefore, the elimination of this sequence during chromosomal evolution could be a mechanism that provides karyotypic stability^64–67^ and might explain its absence in the rearranged autosomes of *O. auyantepui*, while its presence in the Neo-X makes the latter prone to other chromosomal rearrangements, despite providing stability against X-inactivation of autosomal segments^16,17^.

**Figure 8:**
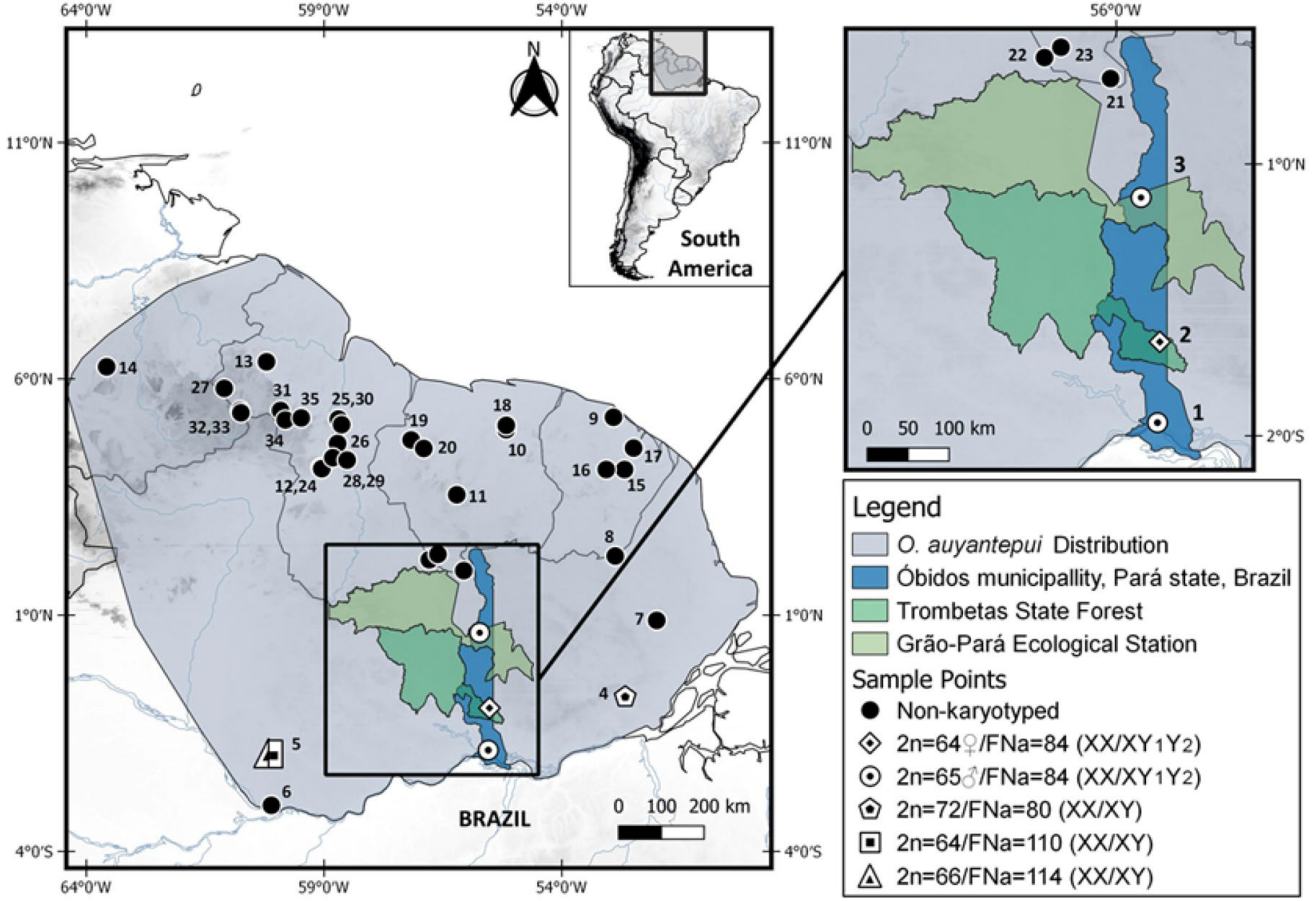
Map showing the distribution, study area and sampling points for *Oecomys auyantepui* specimens analysed in the present study and from literature. Available karyotypic information is shown with description of 2n (diploid number), FNa (autosomal fundamental number), and if is associated with simple (XX/XY) or multiple (XX/XY_1_Y_2_) sex system. Each symbol indicates the karyotype obtained from the locality; more than one symbol indicates that distinct karyotypes were collected at the same locality. Geographic limits of *O. auyantepui* are based on sample points provided in the present study (localities 1–3), and from literature: locality 4^52^, locality 5^53^, localities 6–14^39^, and from GenBank (localities 15–35). The localities mentioned are: Brazil: Pará, Óbidos, forest fragment 7 km distant from the town’s center (locality 1); Trombetas State Forest (locality 2); Grão-Pará Ecological Station (locality 3); Jari River (locality 4); Amazonas, Jatapú River (locality 5); 80 km North of Manaus (locality 6); Amapá, Serra do Navio (locality 7). French Guiana: Trois Sauts (locality 8); Paracou (locality 9). Suriname: Brokopondo, Brownsberg Nature Park (locality 10); Sipaliwini, Avanavero Falls, Kabalebo River (locality 11). Guyana: Upper Takutu–Upper Essequibo (locality 12), Cuyuni-Mazaruni, Kartabo, Cuyuni River (locality 13). Venezuela: Bolívar, Auyan-tepuí, South slope, Río Caroni (locality 14). Samples obtained from GenBank (localities 15–35) are detailed in Supplementary Table 1.

We noticed that the other cytotypes found in *O. auyantepui* (2n = 64, 66 and 72) from the Jatapú and Jari Rivers (Fig. 8, localities 5 and 4, respectively) exhibited distinct morphologies of the X chromosomes (medium submetacentric, large metacentric, and large submetacentric, respectively). Although they are found within a simple sex determination system (XX/XY), the X chromosomes had euchromatic short arms and repetitive sequences at the centromere, such as an ITS in the karyotypes with 2n = 64, 66^52^. Perhaps these differences in size and morphology, plus the presence of ITS, could be a clue to a more complex rearrangement of the X chromosome during its evolution.

Investigations carried out in some groups with multiple sex systems show that the chromosomal evolution in the Neo-X and/or Neo-Y gives rise to other derived systems. This is described in Stenodermatinae bats^68^, in which chromosome painting revealed that a XY_1_Y_2_ system originated a Neo-XY system, due to a translocation between Y_1_ and Y_2_; this Neo-XY has then diverged into two more derivate systems: in one branch, a fission in the Neo-X created a X_1_X_1_X_2_X_2_/X_1_X_2_Y; while in the other branch, a fusion between an autosome and the Neo-Y originated a Neo-X_1_X_1_X_2_X_2_/X_1_X_2_Y. In rodents from the genus *Reithrodon* (Sigmodontinae, Reithrodontini), the Uruguay population exhibits a XY_1_Y_2_ system, while the Brazil population shows a Neo-XY system^44^; a hypothetical intermediate Neo-X/Y_1_Y_2_ formula was the ancestor for the Uruguayan form, while the Brazilian form originated through a fusion between the Y_1_ and Y_2_, that gave rise to the Neo-XY system^44^. The evolutionary process and specific events responsible for the variation in size and morphology of the X chromosomes in the *O. auyantepui* cytotypes (2n = 64, 66 and 72)^52,53^ will be elucidated only after the employment of HME whole chromosome probes.

Taking into consideration the phylogenetic relationships and karyotypic data within *O. auyantepui*, the COI phylogeny is the only one that includes all karyotyped samples for this species from both the present study (2n = 64♀65♂) and the literature (2n = 64, 66 and 72)^52,53^. Despite being considered as a valuable tool for highlighting cases requiring systematic attention among small mammal species^69^, our COI sequences of *O. auyantepui* formed a polytomy with only a few subclades mostly with no satisfactory support (Fig. 4). In fact, specimens of *O. auyantepui* with the multiple sex system grouped in a separated branch only in the concatenated Cytb + FGB-I7 topologies, but with low support in both BI and ML analyses (Fig. 6). Although our molecular analyses do not exhibit a better resolution in the terminal branches, the karyotypic information indicates that we are dealing with at least two distinct lineages. We have three karyotypes with a simple sex system XX/XY (2n = 64, 66 and 72) that could represent variation within a single lineage, while the other lineage corresponds to specimens with a multiple sex system XX/XY_1_Y_2_ (2n = 64♀65♂).

The fact that four potentially karyotypic variants are in a small distribution area (Localities 1–4, Fig. 8) indicates that isolating mechanisms are operating, and the rise of a multiple sex determination system may be acting as a post-zygotic barrier between these apparently sympatric populations, since the difference in sex determination systems will generate aberrations in meiotic synapsis. Taking into consideration the low level of genetic divergence within *O. auyantepui* (mean p-distance 2.15%; Table 2) and the impossibility of interbreeding between these two sex systems, it is most likely that a speciation process is already on course. Similar results were observed in *Deltamys* rodents (Sigmodontinae, Akodontini) where the two distinct sex determination systems (XX/XY and X_1_X_1_X_2_X_2_/X_1_X_2_Y) act as a reproductive barrier that is reflected in the phylogenetic divergence of 11.13–12.14% between the two divergent lineages^42^. Despite the low level of genetic divergence in *O. auyantepui* mentioned above, it is worthy of note that the specimen N228 exhibited 5.20% of mean genetic divergence from other conspecifics included in the Cytb topology (Fig. 5). Further studies are necessary to evaluate if this individual represents a new species, which may be either cytogenetically or morphologically distinct from its closely related *O. auyantepui* individuals.

Some authors have proposed that changes in the sex determination system can alter behavior patterns and contribute to pre-zygotic isolation mechanisms, as described in threespine stickleback fish *Gasterosteus aculeatus*^70^, in which populations with XX/XY and X_1_X_1_X_2_X_2_/X_1_X_2_Y systems exhibit different courtship behavior. In the rodent *Mus minutoides* (Muridae), it was observed that XY females have more reproductive success than XX females and are more aggressive and have a stronger bite than XY males^71^. Thus, differences in the X chromosomes of *O. auyantepui* could lead to behavioral modifications and act in pre-zygotic isolation mechanisms between these two taxonomic entities.

An evaluation of the genetic (Cytb) structure of *Oecomys* aff*. roberti* (= *O. tapajinus*) populations observed isolated and stable populations, but with no influence from the mid-Araguaia River opposite banks^72^. Two sympatric *O. paricola* “eastern clade” populations (OPA-A and OPA-B) from the Belém area of endemism exhibited distinct karyotypes^50^. Both works reached similar conclusions for their respective analyzed species, proposing that *Oecomys* taxa can exhibit isolated populations in the absence of strong geographic barriers.

As discussed above, the fact that four potentially karyotypic variants of *O. auyantepui* are in a small distribution area (Localities 1–4, Fig. 8) could be explained by this type of populational structure for *Oecomys* taxa^50,72^. In this scenario, the rise of a rearranged chromosomal form within an isolated population could be stablished in a few generations, as rodents exhibit a tendency to be organized in demes^73,74^ with low interbreeding among distinct populations^35^. The reproductive barrier would be more intense between the ancestral (XX/XY) and derived (XX/XY_1_Y_2_) systems, as the hybrid offspring would be infertile^6^. We propose that we are dealing with a case of chromosomal speciation, triggered by the emergence of a new sex system (XX/XY_1_Y_2_) in isolated *O. auyantepui* populations. Consequently, *O. auyantepui* is a species complex with at least two distinct lineages, which disagrees with the literature data that recover this taxon as a monotypic species *sensu*^48^.

A comparative analysis of the G-banding patterns among the Sigmodontinae Neo-X chromosomes of *O. auyantepui* (present work), *Reithrodon* (Reithrodontini^44^), and *Salinomys delicatus* (Phyllotini^43^) was performed and this revealed that the autosomal portion translocated in the Neo-X exhibits similar G-banding patterns in the three taxa. This suggests that the same autosomal segment is shared among these distinct lineages. Literature data show that distinct genera within groups that have multiple sex systems may also share the same autosome in the sex-autosome translocation, as in primates from genera *Alouatta*^75^ and *Aotus*^76^ and in bats from genera *Artibeus*, and *Uroderma*^77^, *Chiroderma*, *Mesophylla*, and *Vampyriscus*^68^. However, it has been shown that, in rodents from genera *Proechimys* (Echimyidae) and *Nannomys* (Muridae), species within a genus can have different autosomes translocated to the X chromosome^26,35^.

We suggest that the employment of HME whole chromosome probes in *Reithrodon*, *Salinomys delicatus* and *Oecomys auyantepui* with 2n = 64, 66 and 72 will elucidate the origin of Neo-X chromosomes in Sigmodontinae rodents; also, this will shed light on the evolutionary relationships among the four karyotypes of *O. auyantepui*, and clarify if we are dealing with simple (XX/XY) and multiple (XX/XY_1_Y_2_) sex determination systems; or with derived lineages from the XY_1_Y_2_ system.

## Material and methods

### Ethics

The specimens were captured using pitfall traps^78^ and kept stress-free with full access to food and water until their necessary euthanasia that was performed in accordance with animal welfare guidelines established by Brazilian resolution CFMV n.1000/2012. The necessary euthanasia occurred in accordance with animal welfare guidelines established by the Animal Ethics Committee (Comitê de Ética Animal) from Universidade Federal do Pará (Permit 68-2015), which also approved all experimental protocols of this research. The captures were authorized by the Brazilian Environment Department under license (IBAMA 02047.000384/2007-34). JCP has a permanent field permit (number 13248) from “Instituto Chico Mendes de Conservação da Biodiversidade”. The Cytogenetics Laboratory from UFPa has a special permit number 19/2003 from the Ministry of Environment for samples transport and 52/2003 for using the samples for research. All methods are reported in accordance with ARRIVE guidelines (https://arriveguidelines.org/).

### Samples

We studied the karyotypes of three adult samples of *Oecomys auyantepui* from distinct locations in Óbidos municipality, Pará state, Brazil. The wildlife samples were collected according to the following: one male specimen (UFPAM 2027) was collected in a forest 7 km distant from the town’s center (01° 51′ 15″ S, 55° 32′ 53.4″ W), one female sample (MPEG 39,927) was collected at the Trombetas State Forest (00° 57′ 45.97″ S, 55° 31′ 20.28″ W), and one male sample (MPEG 40,457) was collected at the Grão-Pará Ecological Station (00° 37′ 49.01″ N, 55° 43′ 42.60″ W). The specimens were deposited at the zoological collections of Museu Paraense Emílio Goeldii (MPEG) and the Museu de Zoologia da Universidade Federal do Pará (MUFPA). Both institutions are in Belém, Pará state, Brazil.

### Cytogenetics

The metaphase chromosomal preparations were obtained from bone marrow extraction^79^. The slides containing chromosomal preparations underwent C-Banding^80^, G-Banding^81^ and FISH^82^ techniques. The FISH experiments were performed with human telomeric probes (All Telomere, ONCOR), and with 24 whole chromosome painting probes from a female of *Hylaeamys megacephalus* (HME; 2n = 54)^54^; from the 24 HME whole chromosome probes, 21 correspond to one chromosome each (including the X chromosome), while three corresponded to two pairs of chromosomes each (HME (9,10), (13,22) and (16,17).

### Image capture and analysis

We used an Olympus BX41 microscope and a CCD 1300QDS digital camera to obtain digital images from G-banded and C-banded karyotypes, which were analyzed using the GenASIs software v. 7.2.7.34276. The Nikon H550S microscope with a DS-Qi1Mc digital camera captured the FISH images, which were analyzed using the Nis-Elements software. The karyotypes were organized according to the literature^83^. The final images were edited using Adobe Photoshop CS6 software.

### Molecular analysis

We obtained sequences from *Oecomys auyantepui* samples UFPAM 2027 (Field number LTO05) and MPEG 40,457 (field number CN285). We used partial nucleotide sequences of the mitochondrial genes Cytochrome b (Cytb; 801 base pairs) and Cytochrome C Oxidase Subunit I (COI; 657 base pairs), and sequence data from nuclear beta-​fibrinogen intron 7 (FGB-I7; 727 base pairs). We followed the protocols described in^49^ for the extraction, amplification, and sequencing of Cytb and FGB-I7 genes; we also followed the same protocols for the COI gene, with the primers Fish F2 (TCGACTAATCATAAAGATATCGGCAC) and Fish R2 (ACTTCAGGGTGACCGAAGAATCAGAA)^84^. The sequences obtained in the present study are available on GenBank under accession numbers OM927735, OM927739, OM927737 (CN285); OM927736, OM927740, OM927738 (LTO05) (see Supplementary Table 1).

We also used the sequences available on Genbank (http://www.ncbi.nlm.nih.gov/genbank) from the three genes in order to include all COI sequences available in this database for *O. auyantepui* (48 sequences) plus representative COI sequences of *Oecomys* species (14 sequences), totaling 64 sequences in our COI analysis; all Cytb sequences available for *O. auyantepui* (nine sequences) plus representative Cytb sequences of each *Oecomys* species/clade previously recognized^48,49,51^ (43 sequences), totaling 52 sequences in our Cytb analysis; and all FGB-I7 sequences available for *O. auyantepui* (two sequences) plus representative FGB-I7 sequences of each *Oecomys* species/clade recognized^48,51^) (15 sequences), totaling 19 sequences in our concatenated data analysis (Cytb + FGB-I7) (Supplementary Table 1). *Euryoryzomys nitidus*, *Hylaeamys megacephalus* and *Oligoryzomys utiaritensis* were used as outgroup for Cytb and concatenated analyses, and one sequence of *H. megacephalus* for COI analysis (Supplementary Table 1).

The alignment and editing of the Cytb, COI and concatenated (Cytb + FGB-I7) sequences were conducted using the BioEdit Sequence Alignment Editor program, version 7.0.5.3^85^ with ClustalW tool. A search for the best nucleotide substitution model was made using jModeltest 2.1.6 software^86^ on CIPRES platform^87^, which selected TIM2 + I + G for Cytb, GTR + I + G for COI, and TIM2 + I + G and TPM2uf + G for the concatenated Cytb and FGB-I7, respectively.

The phylogenetic reconstructions were made using Bayesian Inference (BI) and Maximum Likelihood (ML) methods. BI run in MrBayes 3.2.7^88^ with four chains. The algorithm was based on 50 million generations, sampled every 1,000 generations. ML reconstruction was obtained using Garli–2.0^89^ with 1,000 bootstrap replicates and majority consensus tree. The tree was displayed and edited in Figtree v. 1.4.3^90^. Branches supports were evaluated on Bayesian posterior probability in BI and by bootstrap in ML. Cytb genetic divergence values (p-distances) for *O. auyantepui* clades (obtained on Cytb analysis) were calculated with MEGA7^91^.

### Map

The map was made using QGIS v. 3.10.7. The shapefiles containing geographic data (elevation, hydrography, and country limits) were obtained from DIVA-GIS^92^, in the link https://www.diva-gis.org/gdata. Geographic limits of *Oecomys auyantepui* are based on sample points provided in the present study (localities 1–3), by Lira^53^ (locality 4), Gomes Junior et al.^52^ (locality 5), Patton et al.^39^ (localities 6–14), and from GenBank (localities 15–35). More detailed information is available in Supplementary Table 1.

## Supplementary Information

Supplementary Figure 1.

Supplementary Figure 2.

Supplementary Figure 3.

Supplementary Table 1.

## Data Availability

The datasets generated and/or analysed during the current study are available in the GenBank repository (https://www.ncbi.nlm.nih.gov/genbank/). All accession numbers supporting the results reported in this article are found in the main text and in the supplementary files.

## Abbreviations

mtDNA: Mitochondrial DNA
nuDNA: Nuclear DNA
Cytb: Cytochrome b
COI: Cytochrome C Oxidase Subunit I
FGB-I7: Beta-fibrinogen intron 7
BI: Bayesian Inference
ML: Maximum Likelihood
FISH: Fluorescence In Situ Hybridization
ITS: Interstitial telomeric sequence
2n: Diploid number
FNa: Autosomal fundamental number
CH: Constitutive heterochromatin
het-ITS: Heterochromatic-ITS
APRT: Adenosine phosphoribosyltransferase
HSA: Human whole chromosome probes
MMU: *Mus musculus*
HME: *Hylaeamys megacephalus*
OAU: *Oecomys auyantepui*
OCA-PA: *Oecomys catherinae* From Pará
OCA-RJ: *Oecomys catherinae* From Rio de Janeiro
OPA-A: *Oecomys paricola* Cytotype A
OPA-B: *Oecomys paricola* Cytotype B
OPA-C: *Oecomys paricola* Cytotype C
CLA: *Cerradomys langguthi*
NVO: *Neacomys* *vossi*
NEL: *Neacomys* *elieceri*
NXI: *Neacomys* *xingu*
NMA: *Neacomys* *marajoara*
NPA: *Neacomys paracou*
NSP-E: *Neacomys* Sp. E
NAM: *Neacomys amoenus*
TNI: *Thaptomys nigrita*
AMO: *Akodon montensis*
ASP: *Akodon* Sp.
NLA: *Necromys lasiurus*
OAM: *Oxymycterus amazonicus*
BBR: *Blarinomys breviceps*

## References

[1] Bush GL, Case SM, Wilson AC, Patton JL (1977). Rapid speciation and chromosomal evolution in mammals. Proc. Natl. Acad. Sci..

[2] King M (1993). Species Evolution.

[3] Rieseberg LH (2001). Chromosomal rearrangements and speciation. Trends Ecol. Evol..

[4] Castiglia R (2013). Sympatric sister species in rodents are more chromosomally differentiated than allopatric ones: Implications for the role of chromosomal rearrangements in speciation. Mammal Rev..

[5] Franchini P (2020). Reconstructing the evolutionary history of chromosomal races on islands: A Genome-Wide Analysis of Natural House Mouse Populations. Mol. Biol. Evol..

[6] Graves JAM (2016). Did sex chromosome turnover promote divergence of the major mammal groups? De novo sex chromosomes and drastic rearrangements may have posed reproductive barriers between monotremes, marsupials and placental mammals. BioEssays.

[7] Romanenko SA (2020). Evolutionary rearrangements of X chromosomes in voles (Arvicolinae, Rodentia). Sci. Rep..

[8] White MJD (1978). Modes of Speciation.

[9] Sites JW, Moritz C (1987). Chromosomal evolution and speciation revisited. Syst. Zool..

[10] Coyne JA, Orr HA (1998). The evolutionary genetics of speciation. Philos. Trans. R. Soc. Lond. B..

[11] Searle JB (1998). Speciation, chromosomes, and genomes. Genome Res..

[12] Romanenko SA, Volobouev V (2012). Non-Sciuromorph rodent karyotypes in evolution. Cytogenet. Genome Res..

[13] White MJD (1973). Animal Cytology and Evolution.

[14] Wang B, Xia Y, Song J, Wang W, Tang Y (2013). Case report: Potential speciation in humans involving robertsonian translocations. Biom. Res..

[15] Ashley T (2002). X-Autosome translocations, meiotic synapsis, chromosome evolution and speciation. Cytogenet. Genome Res..

[16] Dobigny G, Ozouf-Costaz C, Bonillo C, Volobouev V (2004). Viability of X-autosome translocations in mammals: An epigenomic hypothesis from a rodent case-study. Chromosoma.

[17] Veyrunes F, Perez J (2018). X inactivation in a mammal species with three sex chromosomes. Chromosoma.

[18] Graves JAM (2006). Sex chromosome specialization and degeneration in mammals. Cell.

[19] Lifschytz E, Lindsey DL (1972). The role of X-chromosome inactivation during spermatogenesis. PNAS.

[20] Sharp AJ, Spotswood HT, Robinson DO, Turner BM, Jacobs PA (2002). Molecular and cytogenetic analysis of the spreading of X inactivation in X;autosome translocations. Hum. Mol. Genet..

[21] Searle AG, Beechey CV, Evans EP, Kirk M (1983). Two new X-autosome translocations in the mouse. Cytogenet. Cell Genet..

[22] Kim JW (2012). Molecular and clinical characteristics of 26 cases with structural Y chromosome aberrations. Cytogenet. Genome Res..

[23] Centofante L, Bertollo LAC, Moreira-Filho O (2006). Cytogenetic characterization and description of an XX/XY_1_Y_2_ sex chromosome system in catfish *Harttia carvalhoi* (Siluriformes, Loricariidae). Cytogenet. Genome Res..

[24] Oliveira EA (2018). Tracking the evolutionary pathway of sex chromosome among fishes: Characterizing the unique XX/XY_1_Y_2_ system in *Hoplias malabaricus* (Teleostei, Characiformes). Chromosoma.

[25] Noronha RCR (2020). Meiotic analyses show adaptations to maintenance of fertility in X_1_Y_1_X_2_Y_2_X_3_Y_3_X_4_Y_4_X_5_Y_5_ system of amazon frog *Leptodactylus pentadactylus* (Laurenti, 1768). Sci. Rep..

[26] Veyrunes F, Catalan J, Sicard B, Robinson TJ, Duplantier JM, Granjon L (2004). Autosome and sex chromosome diversity among the African pygmy mice, subgenus *Nannomys* (Murinae; *Mus*). Chromosome Res..

[27] Aquino CI, Abril VV, Duarte JMB (2013). Meiotic pairing of B chromosomes, multiple sexual system, and Robertsonian fusion in the red brocket deer *Mazama americana* (Mammalia, Cervidae). Genet. Mol. Res..

[28] Kasahara S, Dutrillaux B (1983). Chromosome banding patterns of four species of bats, with special reference to a case of X-autosome translocation. Ann. Genet..

[29] Solari AJ, Pigozzi MI (1994). Fine structure of the XY body in the XY1Y2 trivalent of the bat *Artibeus lituratus*. Chromosome Res..

[30] Noronha RCR (2001). Sex-autosome translocations: Meiotic behavior suggests an inactivation block with permanence of autosomal gene activity in Phyllostomid bats. Caryologia.

[31] Noronha RCR (2004). Meiotic analyses of the sex chromosomes in Carolliinae-Phyllostomidae (Chiroptera): NOR separates the XY_1_Y_2_ into two independent parts. Caryologia.

[32] Parish DA, Vise P, Whman HA, Bull JJ, Baker RJ (2002). Distribution of LINEs and other repetitive elements in the karyotype of the bat *Carollia*: Implications for X-chromosome inactivation. Cytogenet. Genome Res..

[33] Amaral PJS (2013). *Proechimys* (Rodentia, Echimyidae): Characterization and taxonomic considerations of a form with a very low diploid number and a multiple sex chromosome system. BMC Genet..

[34] Rodrigues da Costa MJ (2016). Cryptic species in *Proechimys*
*goeldii* (Rodentia, Echimyidae)? A case of molecular and chromosomal differentiation in allopatric populations. Cytogenet. Genome Res..

[35] Oliveira Da Silva W (2019). Identification of two independent X-autosome translocations in closely related mammalian (*Proechimys*) species. Sci. Rep..

[36] Toder R (1997). Comparative chromosome painting between two marsupials: Origins of an XX/XY_1_Y_2_ sex chromosome system. Mamm. Genome..

[37] Vassart M, Séguéla A, Hayes H (1995). Chromosomal evolution in gazelles. J. Hered..

[38] Dias de Oliveira L (2019). First cytogenetic information for *Lonchothrix emiliae* and taxonomic implications for the genus taxa *Lonchothrix* + *Mesomys* (Rodentia, Echimyidae, Eumysopinae). PLoS ONE.

[39] Patton JL, Pardiñas UFJ, D’Elía G (2015). Mammals of South America: Rodents.

[40] Pardiñas UFJ, Teta P, Salazar-Bravo J (2015). A new tribe of sigmodontinae rodents (Cricetidae). Mastoz Neot..

[41] Gonçalves PR (2020). Unraveling deep branches of the sigmodontinae tree (Rodentia: Cricetidae) in Eastern South America. J. Mamm. Evol..

[42] Ventura K, Fagundes V, D’Elía G, Christoff AU, Yonenaga-Yassuda Y (2011). A new allopatric lineage of the rodent *Deltamys* (Rodentia: Sigmodontinae) and the chromosomal evolution in *Deltamys kempi* and *Deltamys* sp.. Cytogenet. Genome Res..

[43] Lanzone C (2011). XY_1_Y_2_ chromosome system in *Salinomys delicatus* (Rodentia, Cricetidae). Genetica.

[44] Ortells MO, Reig AO, Brum-Zorrilla N, Scaglia AO (1988). Cytogenetics and karyosystematics of phyllotine rodents (Cricetidae, Sigmodontinae) I. Chromosome multiformity and gonosomai-autosomai transiocation in Reithrodon. Genetica.

[45] Rosa CC (2012). Genetic and morphological variability in South American rodent *Oecomys* (Sigmodontinae, Rodentia): Evidence for a complex of species. J. Genet..

[46] Pardiñas UFJ, Teta P, Salazar-Bravo J, Myers P, Galliari CA (2016). A new species of arboreal rat, genus *Oecomys* (Rodentia, Cricetidae) from Chaco. J. Mamm..

[47] Malcher SM (2017). *Oecomys catherinae* (Sigmodontinae, Cricetidae): Evidence for chromosomal speciation?. PLoS ONE.

[48] Suárez-Villota EY, Carmignotto AP, Brandão MV, Percequillo AR, Silva MJDJ (2018). Systematics of the genus *Oecomys* (Sigmodontinae: Oryzomyini): Molecular phylogenetic, cytogenetic and morphological approaches reveal cryptic species. Zool. J. Linn. Soc..

[49] Saldanha J (2019). Genetic diversity of *Oecomys* (Rodentia, Sigmodontinae) from the Tapajós River basin and the role of rivers as barriers for the genus in the region. Mamm. Biol..

[50] Oliveira Da Silva W (2020). Karyotypic divergence reveals that diversity in the *Oecomys paricola* complex (Rodentia, Sigmodontinae) from eastern Amazonia is higher than previously thought. PLoS ONE.

[51] Saldanha J, Rossi RV (2021). Integrative analysis supports a new species of the *Oecomys catherinae* complex (Rodentia, Cricetidae) from Amazonia. J. Mamm..

[52] Gomes Júnior RG (2016). Intense genomic reorganization in the genus *Oecomys* (Rodentia, Sigmodontinae): Comparison between DNA barcoding and mapping of repetitive elements in three species of the Brazilian Amazon. Comp. Cytogenet..

[53] Lira, T. *Citogenética clássica e molecular de alguns representantes da tribo Oryzomyini (Rodentia, Cricetidae) da Amazônia Central.* Ph.D dissertation, Universidade Federal do Amazonas. Manaus, Amazonas, Brazil (2012).

[54] Nagamachi CY (2013). FISH with whole chromosome and telomeric probes demonstrates huge karyotypic reorganization with ITS between two species of Oryzomyini (Sigmodontinae, Rodentia): *Hylaeamys megacephalus* probes on *Cerradomys langguthi* karyotype. Chromosome Res..

[55] Suárez P (2015). Clues on syntenic relationship among some species of Oryzomyini and Akodontini Tribes (Rodentia: Sigmodontinae). PLoS ONE.

[56] Pereira AL (2016). Extensive chromosomal reorganization among species of New World muroid rodents (Cricetidae, Sigmodontinae): Searching for phylogenetic ancestral traits. PLoS ONE.

[57] Oliveira Da Silva W (2017). Chromosomal diversity and molecular divergence among three undescribed species of *Neacomys* (Rodentia, Sigmodontinae) separated by Amazonian rivers. PLoS ONE.

[58] Oliveira Da Silva W (2019). Chromosomal phylogeny and comparative chromosome painting among *Neacomys* species (Rodentia, Sigmodontinae) from eastern Amazonia. BMC Evol. Biol..

[59] Oliveira Da Silva W (2020). Chromosomal signatures corroborate the phylogenetic relationships within Akodontini (Rodentia, Sigmodontinae). Int. J. Mol. Sci..

[60] Patton JL, Silva MN, Malcolm JR (2000). Mammals of the Rio Juruá and the Evolutionary and ecological diversification of Amazonia. B Am. Mus. Nat. Hist..

[61] Langguth A, Maia V, Mattevi M (2005). Karyology of large size Brazilian species of the genus *Oecomys* Thomas, 1906 (Rodentia, Muridae, Sigmodontinae). Arq. Mus. Nac. Rio de Janeiro.

[62] Romanenko SA, Perelman PL, Trifonov VA, Graphodatsky AS (2012). Chromosomal evolution in Rodentia. Heredity.

[63] Rahn MI (2016). Protein markers of synaptic behavior and chromatin remodeling of the neo-XY body in phyllostomid bats. Chromosoma.

[64] Aksenova AY, Mirkin SM (2019). At the beginning of the end and in the middle of the beginning: Structure and maintenance of telomeric DNA repeats and interstitial telomeric sequences. Genes.

[65] Kilburn AE, Shea MJ, Sargent RG, Wilson JH (2001). Insertion of a telomere repeat sequence into a mammalian gene causes chromosome instability. Mol. Cell Biol..

[66] Ruiz-Herrera A, Nergadze SG, Santagostino M, Giulotto E (2008). Telomeric repeats far from the ends: Mechanisms of origin and role in evolution. Cytogenet. Genome Res..

[67] Meyne J (1990). Distribution of non-telomeric sites of the (TTAGGG)n telomeric sequence in vertebrate chromosomes. Chromosoma.

[68] Gomes AJB (2016). Chromosomal phylogeny of Vampyressine bats (Chiroptera, Phyllostomidae) with description of two new sex chromosome systems. BMC Evol. Biol..

[69] Borisenko AV, Lim BK, Ivanova NV, Hanner RH, Hebert PD (2008). DNA barcoding in surveys of small mammal communities: A field study in Suriname. Mol. Ecol. Resour..

[70] Kitano J (2009). A role for a neo-sex chromosome in stickleback speciation. Nature.

[71] Ginot S, Claude J, Perez J, Veyrunes F (2017). Sex reversal induces size and performance differences among females of the African pygmy mouse, *Mus*
*minutoides*. J. Exp. Biol..

[72] Rocha RG (2014). Seasonal flooding regime and ecological traits influence genetic structure of two small rodents. Ecol. Evol..

[73] Gilmour J, Gregor J (1939). Demes: A suggested new terminology. Nature.

[74] Patton JL, Sherwood SW (1983). Chromosome evolution and speciation in rodents. Ann. Rev. Ecol. Syst..

[75] de Oliveira EHC (2002). The phylogeny of howler monkeys (*Alouatta*, Platyrrhini): Reconstruction by multicolor cross-species chromosome painting. Chromosome Res..

[76] Araújo NP, Stanyon R, Pereira VS, Svartman M (2019). Interspecific chromosome painting provides clues to the ancestral karyotype of the New World monkey genus *Aotus*. J. Mamm. Evol..

[77] Pieczarka JC (2013). A phylogenetic analysis using multidirectional chromosome painting of three species (*Uroderma*
*magnirostrum*, *U.*
*bilobatum* and *Artibeus*
*obscurus*) of subfamily Stenodermatinae (Chiroptera-Phyllostomidae). Chromosome Res..

[78] Corn PS, Heyer WR, Donnelly MA, McDiarmid RW, Hayek LC, Foster MS (1994). Straight-line drift fences and pitfall traps. Measuring and Monitoring Biological Standard Methods for Amphibians.

[79] Ford CE, Hamerton JL (1956). A colchicine, hypotonic-citrate, squash sequence for mammalian chromosomes. Stain Technol..

[80] Sumner AT (1972). A simple technique for demonstrating centromeric heterochromatin. Exp. Cell Res..

[81] Sumner AT, Evans HJ, Buckland RA (1971). New technique for distinguishing between human chromosomes. Nat. Lond. New Biol..

[82] Yang F, Carter NP, Shi L, Ferguson-Smith MA (1995). A comparative study of karyotypes of muntjacs by chromosome painting. Chromosoma.

[83] Levan A, Fredga K, Sandberg AA (1964). Nomenclature for centromeric position on chromosomes. Hereditas.

[84] Ward RD, Zemlak TS, Innes BH, Last PR, Hebert PDN (2005). DNA barcoding Australia’s fish species. Philos. Trans. R. Soc. Lond. B Biol. Sci..

[85] Hall TA (1999). BioEdit: A user-friendly biological sequence alignment editor and analysis program for Windows 85/98/NT. Nucleic Acids Symp. Ser..

[86] Darriba D, Taboada GL, Doallo R, Posada D (2012). jModelTest 2: More models, new heuristics and parallel computing. Nat. Methods..

[87] Miller, M. A., Pfeiffer, W. & Schwartz, T. Creating the CIPRES Science Gateway for inference of large phylogenetic trees. *Proceedings of the Gateway Computing Environments Workshop (GCE) IEE*, New Orleans, LA, 1–8 (2010). 10.1109/GCE.2010.5676129

[88] Ronquist F (2012). MrBayes 3.2: Efficient Bayesian phylogenetic inference and model choice across a large model space. Syst. Biol..

[89] Zwickl, D. J. *Genetic algorithm approaches for the phylogenetic analysis of large biological sequence datasets under the maximum likelihood criterion*. Ph.D. dissertation, The University of Texas at Austin. Austin, Texas (2016).

[90] Rambaut, A. *FigTree v1.4.3 2006–2016*. http://tree.bio.ed.ac.uk/software/figtree/ (2016).

[91] Kumar S, Stecher G, Tamura K (2016). MEGA7: Molecular evolutionary genetics analysis version 7.0 for bigger datasets. Mol. Biol. Evol..

[92] Hijmans, R. J. et al. DIVA-GIS. *Vsn 5.0. A geographic information system for the analysis of species distribution data*. http://www.diva-gis.org (2004).

