# Supplementary material for: The emergence of a new sex-system (XX/XY1Y2) suggests a species complex in the “monotypic” rodent Oecomys auyantepui (Rodentia, Sigmodontinae)
Source: Sci Rep. 2022 May 24;12:8690. doi: 10.1038/s41598-022-12706-3 (PMC9130129; doi:10.1038/s41598-022-12706-3)
Supplement: Supplementary file 4 — Supplementary Table 1. [file 41598_2022_12706_MOESM4_ESM.docx]

The emergence of a new sex-system (XX/XY_1_Y_2_) suggests a species complex in the “monotypic” rodent *Oecomys auyantepui* (Rodentia, Sigmodontinae)

**Willam Oliveira da Silva^1^ 0000-0003-3125-1075, Celina Coelho Rosa^1^, Malcolm Andrew Ferguson-Smith^2^ 0000-0001-9372-1381, Patricia Caroline Mary O’Brien^2^, Juliane Saldanha^3^ 0000-0003-3983-7169, Rogério Vieira Rossi^3^ 0000-0003-2353-5000, Julio Cesar Pieczarka^1^# 0000-0003-2951-8877, Cleusa Yoshiko Nagamachi^1^#* 0000-0003-1516-2734**

^1^ Laboratório de Citogenética, Centro de Estudos Avançados da Biodiversidade, Instituto de Ciências Biológicas, Universidade Federal do Pará (UFPA), Belém, Pará, Brazil.

^2^ Cambridge Resource Centre for Comparative Genomics, Department of Veterinary Medicine, University of Cambridge, Cambridge, UK.

^3^ Departamento de Biologia e Zoologia, Instituto de Biociências, Universidade Federal do Mato Grosso (UFMT), Mato Grosso, Brazil.

***Correspondence:** cleusanagamachi@gmail.com

**Supplementary Fig. 1** FISH of *Oecomys auyantepui* (OAU) metaphases with *Hylaeamys megacephalus* (HME) whole chromosome probes. For each metaphase, the hybridized probes can be shown as green (FITC) or red (CY3) and are noted in the same color (top right); OAU chromosomes hybridized with HME probes are noted in white; the same metaphase is shown with DAPI staining (blue) on the left. The hybridized probes were HME 1-8, (9,10), 11, 12, (13,22) and X.

**Supplementary Fig. 2** FISH of *Oecomys auyantepui* (OAU) metaphases with *Hylaeamys megacephalus* (HME) whole chromosome probes. For each metaphase, the hybridized probes can be shown as green (FITC) or red (CY3) and are noted in the same color (top right); OAU chromosomes hybridized with HME probes are noted in white; the same metaphase is shown with DAPI staining (blue) on the left. The hybridized probes were HME 14, 15, (16,17), 18-21, 23-26.

**Supplementary Fig. 3** Idiograms of the karyotypes of *Oecomys auyantepui* (OAU), *O. paricola* cytotype A (OPA-A), *O. paricola* cytotype B (OPA-B), *O. paricola* cytotype C (OPA-C), *O. catherinae* from Pará (OCA-PA), and *O. catherinae* from Rio de Janeiro (OCA-RJ), as assessed based on HME whole chromosome probes [54]. The karyotypic content of chromosomes involved in rearrangements of each species is separated into columns; the syntenic blocks involved in the rearrangements are arranged horizontally. The box contains an idiogram of HME karyotype previously elaborated [58] and adapted in the present study. Each HME chromosome is shown with a single color, except the pairs (9,10), (13,22) and (16,17), which have one color each. (H) indicates large block of constitutive heterochromatin.

**Supplementary Table 1.** List of sequenced specimens included in the molecular analysis of Cytochrome b (*Cyt*b), Nuclear Beta-Fibrinogen-Intron 7 (FGB-I7), and Cytochrome C Oxidase – Subunit I (COI) in the present study. For each species the Field number or museum acronym, GenBank accession number, collecting locality, latitude (Lat.), longitude (Long.) and references are provided. Brazilian (BR) states are Amazonas (AM), Acre (AC), Mato Grosso (MT), Mato Grosso do Sul (MS), Minas Gerais (MG), Pará (PA), Paraná (PR), Piauí (PI), Rondônia (RO), and São Paulo (SP). BO (Bolivia), EC (Ecuador), FG (French Guiana), GN (Guyana), PE (Peru), and SR (Suriname). Karyotypic information of diploid number (2n) and autosomal fundamental number (FNa) are also provided. Karyotyped specimens in the present study are in bold. The numbers in parentheses refer to the localities shown in Figure 8. Only samples of *Oecomys auyantepui* had their locations numbered and are plotted in Figure 8.

| **Taxon** | **Field Number** | **Voucher Number** | **Karyotype** | ***Cyt*b** | **FGB-I7** | **COI** | **Locality** | **Lat.** | **Long.** | **Reference** |
| --- | --- | --- | --- | --- | --- | --- | --- | --- | --- | --- |
| ***O. auyantepui*** | **CN285** | **MPEG40457** | **2n=65, FNa=84 ♂** | OM927735 | OM927739 | OM927737 | (3) BR, PA: Óbidos: Estação Ecológica Grão-Pará | 0.630 | -55.728 | This study |
| ***O. auyantepui*** | **LTO05** | **UFPAM2027** | **2n=65, FNa=84 ♂** | OM927736 | OM927740 | OM927738 | (1) BR, PA: Óbidos: 7 km from the town’s center | -1.854 | -55.548 | This study |
| ***O. auyantepui*** | **CN120** | **MPEG39927** | **2n=64, FNa=84 ♀** | JF759666 | - | - | (2) BR, PA: Óbidos: Floresta Estadual do Trombetas | -0.962 | -55.522 | Rosa et al. (2012) |
| *O. auyantepui* | V-1001 | - | - | AJ496305 | - | - | (15) FG: Cayenne, Les Nouragues | 4.083 | -52.683 | Mauffrey et al. (unpubl.) |
| *O. auyantepui* | V-971 | - | - | AJ496304 | - | - | (15) FG: Cayenne, Les Nouragues | 4.083 | -52.683 | Mauffrey et al. (unpubl.) |
| *O. auyantepui* | - | MNHN-1994.124 | - | AJ496303 | - | - | (16) FG: Cayenne, Saint-Eugene | 4.083 | -52.683 | Mauffrey et al. (unpubl.) |
| *O. auyantepui* | N228 | - | - | AJ493619 | - | - | (15) FG: Les Nouragues | - |  | Mauffrey et al. (unpubl.) |
| *O. auyantepui* | ROM113975 | - | - | KP778200 | - | - | (18) SR: Brokopondo, Brownsberg Nature Park, Jeep Trail | 5.018 | -55.169 | Almendra et al. (unpubl.) |
| *O. auyantepui* | ROM114316 | - | - | KP778287 | KP778664 | JQ601049 | (18) SR: Brokopondo, Brownsberg Nature Park, Jeep Trail | 5.018 | -55.169 | Almendra et al. (unpubl.) |
| *O. auyantepui* | ROM114059 | - | - | KP778302 | KP778670 | - | (18) SR: Brokopondo, Brownsberg Nature Park, Jeep Trail | 5.018 | -55.169 | Almendra et al. (unpubl.) |
| *O. auyantepui* | ROM114146 | - | - | KP778332 | - | - | (18) SR: Brokopondo, Brownsberg Nature Park, Jeep Trail | 5.018 | -55.169 | Almendra et al. (unpubl.) |
| *O*. *auyantepui* | M24 | INPA6751 | 2n=66, FNa=114 ♂ | - | - | KT258613 | (4) BR, AM: Jatapu river | -1.917 | -60.167 | Gomes Junior et al. (2016) |
| *O*. *auyantepui* | M27 | INPA6753 | - | - | - | KT258614 | (4) BR, AM: Jatapu river | -1.917 | -60.167 | Gomes Junior et al. (2016) |
| *O*. *auyantepui* | M28 | INPA6754 | 2n=64, FNa=110 ♂ | - | - | KT258615 | (4) BR, AM: Jatapu river | -1.917 | -60.167 | Gomes Junior et al. (2016) |
| *O*. *auyantepui* | M517 | INPA6777 | - | - | - | KT258611 | (4) BR, AM: Jatapu river | -1.917 | -60.167 | Gomes Junior et al. (2016) |
| *O*. *auyantepui* | TAG3165 | INPA5225 | 2n=72, FNa=80 | - | - | KT258612 | (5) BR, PA: Jari river | -0.712 | -52.667 | Lira (2012) |
| *O*. *auyantepui* | ROM102944 | - | - | - | - | EU095447 | (24) GN: Upper Takutu-Upper Essequibo | 4.100 | -59.050 | Borisenko et al. (2008) |
| *O*. *auyantepui* | ROM103051 | - | - | - | - | JF491639 | (24) GN: Upper Takutu-Upper Essequibo 5 Km SE of Surama | 4.100 | -59.050 | Engstrom et al. (unpubl.) |
| *O*. *auyantepui* | ROM103052 | - | - | - | - | JF491622 | (24) GN: Upper Takutu-Upper Essequibo 5 Km SE of Surama | 4.100 | -59.050 | Engstrom et al. (unpubl.) |
| *O*. *auyantepui* | ROM103244 | - | - | - | - | JF491633 | (24) GN: Upper Takutu-Upper Essequibo 5 Km SE of Surama | 4.100 | -59.050 | Engstrom et al. (unpubl.) |
| *O*. *auyantepui* | ROM103245 | - | - | - | - | JF491631 | (24) GN: Upper Takutu-Upper Essequibo 5 Km SE of Surama | 4.100 | -59.050 | Engstrom et al. (unpubl.) |
| *O*. *auyantepui* | ROM103288 | - | - | - | - | JF491640 | (24) GN: Upper Takutu-Upper Essequibo 5 Km SE of Surama | 4.100 | -59.050 | Engstrom et al. (unpubl.) |
| *O*. *auyantepui* | ROM103433 | - | - | - | - | JF491626 | (25) GN: Upper Demerara-Berbice, Tropenbos | 5.150 | -58.700 | Engstrom et al. (unpubl.) |
| *O*. *auyantepui* | ROM103502 | - | - | - | - | JF491629 | (25) GN: Upper Demerara-Berbice, Tropenbos | 5.150 | -58.700 | Engstrom et al. (unpubl.) |
| *O*. *auyantepui* | ROM107124 | - | - | - | - | JF491632 | (26) GN: Potaro-Siparuni, Iwokrama Reserve, Giaconda Camp | 4.633 | -58.717 | Engstrom et al. (unpubl.) |
| *O*. *auyantepui* | ROM108236 | - | - | - | - | JF491635 | (27) GN: Cuyuni-Mazaruni, Namai Creek, 5 Km W of Puruima | 5.800 | -61.100 | Engstrom et al. (unpubl.) |
| *O*. *auyantepui* | ROM108691 | - | - | - | - | JF491627 | (28) GN: Potaro-Siparuni, Cow Fly Camp, Iwokrama Reserve | 4.333 | -58.817 | Engstrom et al. (unpubl.) |
| *O*. *auyantepui* | ROM111579 | - | - | - | - | JF491637 | (29) GN: Potaro-Siparuni, Kabukalli Landing, Iwokrama Forest | 4.333 | -58.817 | Engstrom et al. (unpubl.) |
| *O*. *auyantepui* | ROM111620 | - | - | - | - | JF491630 | (29) GN: Potaro-Siparuni, Kabukalli Landing, Iwokrama Forest | 4.333 | -58.817 | Engstrom et al. (unpubl.) |
| *O*. *auyantepui* | ROM113415 | - | - | - | - | JF491641 | (30) GN: Upper Demerara-Berbice, West Pibiri, Mabura | 5.033 | -58.633 | Engstrom et al. (unpubl.) |
| *O*. *auyantepui* | ROM114323 | - | - | - | - | JQ601051 | (18) SR: Brownsberg Nature Park, Jeep Trail | 4.933 | -55.200 | iBOL |
| *O*. *auyantepui* | ROM114331 | - | - | - | - | JQ601054 | (18) SR: Brownsberg Nature Park, Jeep Trail | 4.933 | -55.200 | iBOL |
| *O*. *auyantepui* | ROM114338 | - | - | - | - | JQ601056 | (18) SR: Brownsberg Nature Park, Jeep Trail | 4.933 | -55.200 | iBOL |
| *O*. *auyantepui* | ROM114342 | - | - | - | - | JQ601058 | (18) SR: Brownsberg Nature Park, Jeep Trail | 4.950 | -55.183 | iBOL |
| *O*. *auyantepui* | ROM114357 | - | - | - | - | JQ601060 | (18) SR: Brownsberg Nature Park, Jeep Trail | 4.933 | -55.200 | iBOL |
| *O*. *auyantepui* | ROM114358 | - | - | - | - | JQ601061 | (18) SR: Brownsberg Nature Park, Jeep Trail | 4.933 | -55.200 | iBOL |
| *O*. *auyantepui* | ROM114631 | - | - | - | - | JF491638 | (31) GN: Potaro-Siparuni, Mount Ayanganna, Toe Slope Camp | 5.333 | -59.917 | Engstrom et al. (unpubl.) |
| *O*. *auyantepui* | ROM115081 | - | - | - | - | JF491625 | (32) GN: Cuyuni-Mazaruni, Mount Roraima, Ridge Camp | 5.333 | -60.767 | Engstrom et al. (unpubl.) |
| *O*. *auyantepui* | ROM115082 | - | - | - | - | JF491624 | (32) GN: Cuyuni-Mazaruni, Mount Roraima, Ridge Camp | 5.333 | -60.767 | Engstrom et al. (unpubl.) |
| *O*. *auyantepui* | ROM115097 | - | - | - | - | JF491634 | (32) GN: Cuyuni-Mazaruni, Mount Roraima, Ridge Camp | 5.333 | -60.767 | Engstrom et al. (unpubl.) |
| *O*. *auyantepui* | ROM115156 | - | - | - | - | JF491628 | (33) GN: Cuyuni-Mazaruni, Mount Roraima, Second Camp | 5.283 | -60.750 | Engstrom et al. (unpubl.) |
| *O*. *auyantepui* | ROM115788 | - | - | - | - | JF491636 | (34) GN: Potaro-Siparuni, Mount Wokomung | 5.133 | -59.817 | Engstrom et al. (unpubl.) |
| *O*. *auyantepui* | ROM116688 | - | - | - | - | JF491623 | (35) GN: Potaro-Siparuni, Kaieteur National Park, Kaieteur Falls | 5.175 | -59.481 | Engstrom et al. (unpubl.) |
| *O*. *auyantepui* | ROM117076 | - | - | - | - | JF491621 | (20) SR: Nickerie, Sipaliwini, Bakhuis, Transect 7 | 4.529 | -56.904 | Engstrom et al. (unpubl.) |
| *O*. *auyantepui* | ROM117347 | - | - | - | - | EU096817 | (19) SR: Sipaliwini | 4.710 | -57.170 | Borisenko et al. (2008) |
| *O*. *auyantepui* | ROM117349 | - | - | - | - | EU096820 | (19) SR: Sipaliwini | 4.660 | -57.180 | Borisenko et al. (2008) |
| *O*. *auyantepui* | ROM117495 | - | - | - | - | EU096819 | (19) SR: Sipaliwini | 4.470 | -56.910 | Borisenko et al. (2008) |
| *O*. *auyantepui* | ROM117634 | - | - | - | - | EU096818 | (19) SR: Sipaliwini | 4.810 | -56.990 | Borisenko et al. (2008) |
| *O*. *auyantepui* | ROMMAM120145 | - | - | - | - | HQ545608 | (21) SR: Sipaliwini | 1.944 | -56.063 | iBOL |
| *O*. *auyantepui* | ROMMAM120164 | - | - | - | - | HQ545625 | (21) SR: Sipaliwini | 1.944 | -56.063 | iBOL |
| *O*. *auyantepui* | ROMMAM120183 | - | - | - | - | HQ545642 | (19) SR: Sipaliwini, Kushere Landing, Sipaliwini River | 1.944 | -56.063 | Lim et al. (unpubl.) |
| *O*. *auyantepui* | ROMMAM120546 | - | - | - | - | JQ601162 | (22) SR: Kutari River Camp | 2.175 | -56.788 | iBOL |
| *O*. *auyantepui* | ROMMAM120547 | - | - | - | - | JQ601163 | (22) SR: Kutari River Camp | 2.175 | -56.788 | iBOL |
| *O*. *auyantepui* | ROMMAM120583 | - | - | - | - | JQ601188 | (22) SR: Kutari River Camp | 2.175 | -56.788 | iBOL |
| *O*. *auyantepui* | ROMMAM120632 | - | - | - | - | JQ601224 | (23) SR: Sipaliwini River Camp | 2.289 | -56.607 | iBOL |
| *O*. *auyantepui* | ROMMAM120689 | - | - | - | - | JQ601277 | (23) SR: Sipaliwini River Camp | 2.289 | -56.607 | iBOL |
| *O*. *auyantepui* | V2790 | - | - | - | - | KM102738 | (17) FG: Cacao | 4.538 | -52.491 | Lavergne et al. (2015) |
| *"O*. *auyantepui"* | ROM119775 | - | - | - | - | JF459219 | GN: Potaro-Siparuni, Iwokrama Forest, Canopy Walkway | 4.250 | -58.909 | Lim et al. (unpubl.) |
| *O. bicolor* | ROM104505 | - | - | - | - | JQ601078 | EC: Parque Nacional Yasuni | - |  | iBOL |
| *O. bicolor* | ROMF41922 | - | - | - | - | JF444362 | EC: Orellana | - |  | Eger et al. (Unpubl.) |
| *O. bicolor* | ROM113707 | - | - | - | - | JF491649 | GN: Demerara-Mahaica | - |  | Engstrom et al. (unpubl.) |
| *O. bicolor* - southern | M97074 | MZUSP29528 | 2n=80, FNa=140 | MG323726 | MG323812 | - | BR, MT: Cláudia | -11.583 | -55.167 | Suárez-Villota et al. (2018) |
| *O. bicolor* - eastern | FSF34r | - |  | KR190445 | - | - | BR, PA: Santana do Araguaia | -9.630 | -50.144 | Rocha et al. (2015) |
| *O. bicolor* - norther | T1695 | AMNH269823 |  | AJ496307 | - | - | FG: Cayenne, Los Nouragues | 4.083 | -52.683 | Mauffrey et al. (unpubl.) |
| *O. bicolor* - westernmost | - | MVZ154988 |  | JQ312122 | - | - | PE: Amazonas, Huampami | -5.516 | -79.816 | Rocha et al. (2012) |
| *O. bicolor -* central* | M968410 | MZUSP29523 | 2n=80, FNa=140 | MG323750 | MG323834 | - | BR, MT: Apiacás | -9.567 | -57.383 | Suárez-Villota et al. (2018) |
| *O. bicolor* - western* | MJJS68 | - | 2n=80, FNa=140 | MG323751 | MG323835 | - | BR, AC: Floresta Estadual do Antimary | -9.333 | -68.317 | Suárez-Villota et al. (2018) |
| *O. catherinae* - central | PCH3998 | - | 2n=60, FNa=62 | MG323764 | MG323846 |  | BR, SP: São Joaquim da Barra | -20.483 | -47.850 | Suárez-Villota et al. (2018) |
| *O. catherinae* - eastern | CIT2096 | - | 2n=60, FNa=62 | MG323772 | MG323774 |  | BR, MG: Parque Estadual do Rio Doce | -19.533 | -42.533 | Suárez-Villota et al. (2018) |
| *O. catherinae* - northern | APC288 | MZUSP35538 | 2n=62, FNa=62 | MG323765 | MG323847 |  | BR, MT: Vila Rica | -10.017 | -51.117 | Suárez-Villota et al. (2018) |
| *O. catherinae* - westernmost | APC243 | MZUSP35535 | 2n=60, FNa=62 | MG323759 | MG323841 | - | BR, MT: Aripuanã | -10.167 | -59.450 | Suárez-Villota et al. (2018) |
| *O. cleberi* - central | PCH3674 | - | 2n=80, FNa=134 | MG323737 | MG323821 |  | BR, SP: Guará | -20.500 | -47.833 | Suárez-Villota et al. (2018) |
| *O. cleberi* - northwestern | APC210 | MZUSP35534 | 2n=80, FNa=140 | MG323745 | MG323829 | - | BR, MT: Aripuanã | -10.167 | -59.450 | Suárez-Villota et al. (2018) |
| *O. concolor* | ROM105321 | - | - | - | - | JF491662 | EC: Napo, Parque Nacional Yasuni | - |  | Engstrom et al. (unpubl.) |
| *O. concolor* | ROMF41879 | - | - | - | - | JF444366 | EC: Orellana | - |  | Eger et al. (Unpubl.) |
| *O. concolor* | ROM106145 | - | - | - | - | JF491619 | EC: Napo, Parque Nacional Yasuni | - |  | Engstrom et al. (unpubl.) |
| *O. concolor* | JLP16728 | - | 2n=60, FNa=62 ^+^ | HM594614 | - |  | BR, AM: Rio jaú | -2.216 | -62.383 | Rocha et al. (2011) |
| *O. franciscorum* | - | LBCE1924 | 2n=72, FNa=90^+^ | AY072710 | - | - | BR, MS: Corumbá | -19.000 | -57.650 | Andrade and Bonvicino (2003) |
| *O. franciscorum* | PNPA300 | MZUSP35540 | - | MG323716 | MG323786 | - | BR, MT: Parque Nacional do Pantanal | -17.650 | -57.433 | Suárez-Villota et al. (2018) |
| *O. mamorae* - eastern |  | MVZ197505 |  | HM594605 | - | - | BR, MT: Poconé | -17.120 | -55.946 | Rocha et al. (2011) |
| *O. mamorae* - southern |  | MSB63355 |  | KT737229 | - | - | BO: Chuquisaca | -19.716 | -63.850 | Pardiñas et al. (2016) |
| *O. mamorae* - western |  | MSB68481 |  | KT737227 | - | - | BO: Beni | -15.116 | -68.866 | Pardiñas et al. (2016) |
| *O. matogrossensis* | APC244 | MZUSP29531 | 2n=54, FNa=54 | MG323756 | MG323777 |  | BR, MT: Aripuanã | -10.167 | -59.450 | Suárez-Villota et al. (2018) |
| *O. paricola* - eastern | UU43 | - | 2n=70, FNa=76 | MG323707 | MG323782 | - | BR, PI: Estação Ecológica de Uruçuí-Uma | -8.867 | -44.967 | Suárez-Villota et al. (2018) |
| *O. paricola* - eastern (cytotype A; Oliveira da Silva et al. 2020) | - | MPEG39699 | 2n=72, FNa=75 | JF759681 | - | - | BR, PA: Belém, Parque Ambiental, Utinga | -1.450 | -48.483 | Rosa et al. (2012) |
| *O. paricola* - northern | - | MPEG40851 | 2n=70, FNa=72, | JF759674 | - | - | BR, PA: Marajó island, Chaves, Tauarí Farm | -0.650 | -50.183 | Rosa et al. (2012) |
| *O. paricola* - western | M97023 | MZUSP29525 | 2n=70, FNa=74 | MG323713 | MG323802 | - | BR, MT: Cláudia | -11.583 | -55.167 | Suárez-Villota et al. (2018) |
| *O. rex* | CSA22 | - | - | MK874428 | - | - | BR, PA | - | - | Saldanha et al. (2019) |
| *O. rex* | ROMMAM120287 | - | - | - | - | HQ919650 | SR | - |  | iBOL |
| *O. rex* | INPA5049 | - | - | - | - | KT258610 | - | - |  | Gomes (Unpubl.) |
| *O. roberti* - western | JLP15241 | MVZ200947 | 2n=80, FNa=114 | U58384 | - | - | BR, AM: Penedo, right bank Rio Juruá | -6.833 | -70.750 | Patton and Da Silva (1995); Patton et al. (2000) |
| *O. roberti* - central | M968464 | - | 2n=82, FNa=106 | MG323719 | MG323806 |  | BR, MT: Apiacás | -9.567 | -57.383 | Suárez-Villota et al. (2018) |
| *O. roberti* - central | - | UFPB494 | 2n=82, FNa=110 | FJ361072 | - | - | BR, RO: UHE Samuel | -8.750 | -63.467 | Miranda et al. (unpubl.) |
| *O. rutilus* | ROM107083 | - | - | - | - | JF491681 | GN: Potaro-Siparuni, Mount Wokomung | - |  | Engstrom et al. (unpubl.) |
| *O. rutilus* | ROM115813 | - | - | - | - | JF491682 | GN: Potaro-Siparuni, Mount Wokomung | - |  | Engstrom et al. (unpubl.) |
| *O. rutilus* | CN123 | - | 2n=54, FNa=82-90^+^ | JF759665 | - | - | BR, PA: Óbidos | -1.166 | -55.650 | Rosa et al. (2012) |
| *O. superans* | - | MVZ200944 | 2n=80, FNa=108^+^ | U58385 | - | - | BR, AM: Penedo, right bank Rio Juruá | -6.833 | -70.750 | Patton and Da Silva (1995); Patton et al. (2000) |
| *O. sydandersoni* | - | USNM588189 |  | KT737235 | - | - | BO: Santa Cruz | -14.766 | -61.033 | Pardiñas et al. (2016) |
| *O. tapajinus* | - | UFES1359 | - | HM594600 | - | - | BR, TO: Lagoa da Confusão | -10.816 | -49.713 | Rocha et al. (2011) |
| *O. tapajinus* | - | JFV329 | - | MF523728 | MF592489 | - | BR, PR | - |  | Rocha et al. (2018) |
| *O. tapajinus* | LPC690 | - | - | - | - | MF523761 | BR: 21 km NW Peixe, Rio Santa Teresa | - |  | Rocha et al. (2018) |
| *O. tapajinus* | LPC689 | - | - | - | - | MF523762 | BR: 22 km NW Peixe, Rio Santa Teresa | - |  | Rocha et al. (2018) |
| *O. trinitatis* | - | MUSM13320 | 2n=58, FNa=96^+^ | GU126527 | - | - | PE: Loreto, Rio Galvez | -5.200 | -72.883 | Percequillo et al. (2011) |
| *Oecomys* sp. 1 |  | MVZ155005 |  | JF693876 |  |  | PE: Loreto, Rio Galvez | -5.516 | -79.816 | Pine et al. (2012) |
| *Oecomys* sp. 2 | JUR354 | MVZ200905 | 2n=86, FNa=98 | U58388 | - | - | BR, AM: Lago Vai-Quem-Quer, right bank Rio Juruá | -3.317 | -66.017 | Patton and Da Silva (1995); Patton et al. (2000) |
| *Oecomys* sp. 3 |  | MSB68480 |  | KT737233 | - | - | BO: Beni | -15.116 | -68.866 | Pardiñas et al. (2016) |
| *Euryoryzomys nitidus* | M968417 | MZUSP29524 | - | MG323695 | MG323787 | - | BR, MT: Apiacás | -9.567 | -57.383 | Suárez-Villota et al. (2018) |
| *Hylaeamys megacephalus* | M968452 | - | - | MG323696 | MG323788 | - | BR, MT: Apiacás | -9.567 | -57.383 | Suárez-Villota et al. (2018) |
| *Oligoryzomys utiaritensis* |  | MN75625 | - | JQ013752 | JQ282893 | - | BR, MT: Sapezal | - |  | Agrellos et al. (2012) |

* Specimen recovered as *O. cleberi* on concatenated analysis.

^+^ Diploid number (2n) and autosomal fundamental number (FNa) information for the species, not for the sample.

Agrellos, R., Bonvicino, C.R., Rosa, E.S.T., Marques, A.A.R., D'Andrea, P.S., Weksler, M. The taxonomic status of the Castelo dos Sonhos Hantavirus reservoir, *Oligoryzomys* *utiaritensis* Allen 1916 (Rodentia: Cricetidae: Sigmodontinae). *Zootaxa*. **2011**, 3220: 1-28. doi: 10.11646/zootaxa.3220.1.1

Andrade, A.F.B., Bonvicino, C.R. A new karyologic variant of *Oecomys* (Rodentia: Sigmodontinae) and its phylogenetic relationship based on molecular data. *Genome*. **2003**, 46: 195–203. doi: 10.1139/g02-123

Borisenko, A.V., Lim, B.K., Ivanova, N.V., Hanner, R.H., Hebert, P.D.N. DNA barcoding in surveys of small mammal communities: a field study in Suriname. *Mol Ecol Resour*. **2008**, 8: 471-479. doi: 10.1111/j.1471-8286.2007.01998.x

Gomes Júnior, R.G., Schneider, C.H., Lira, T., Carvalho, N.D.M., Feldberg, E., da Silva, M.N.F., et al. Intense genomic reorganization in the genus *Oecomys* (Rodentia, Sigmodontinae): comparison between DNA barcoding and mapping of repetitive elements in three species of the Brazilian Amazon. *Comp Cytogenet*. **2016**, 10(3): 401–426. doi: 10.3897/CompCytogen.v10i3.8306

Lavergne, A., de Thoisy, B., Donato, D., Guidez, A., Matheus, S., Catzeflis, F., Lacoste, V. Patawa Virus, a New Arenavirus Hosted by Forest Rodents in French Guiana. *Ecohealth*. **2015**, 12(2): 339-46. doi: 10.1007/s10393-014-0971-6

Lira, T. *Citogenética clássica e molecular de alguns representantes da tribo Oryzomyini (Rodentia, Cricetidae) da Amazônia Central.* Ph.D dissertation, Universidade Federal do Amazonas. Manaus, Amazonas, Brazil (2012)

Oliveira da Silva, W., Rosa, C.C., Pieczarka, J.C., Ferguson-Smith, M.A., O’Brien, P.C.M., Mendes-Oliveira, A.C., et al. Karyotypic divergence reveals that diversity in the *Oecomys paricola* complex (Rodentia, Sigmodontinae) from eastern Amazonia is higher than previously thought. *PLoS ONE.* **2020**, 15(10): e0241495. doi: 10.1371/journal.pone.0241495

Pardiñas, U.F.J., Teta, P., Salazar-Bravo, J., Myers, P., Galliari, C.A. A new species of arboreal rat, genus *Oecomys* (Rodentia, Cricetidae) from Chaco. *J* *Mammal*. **2016**, 97: 1177-1196. Doi: 10.1093/jmammal/gyw070

Patton, J.L., Da Silva, M.N.F. A review of the spiny mouse genus *Scolomys* (Rodentia: Muridae: Sigmodontinae) with the description of a new species from the western Amazon of Brazil. *Proc. Biol. Soc. Wash.* **1995**, 108: 319-337.

Patton, J.L., Silva, M.N., Malcolm, J.R. Mammals of the Rio Juruá and the Evolutionary and ecological diversification of Amazonia. *B Am Mus Nat Hist.* **2000**, 244: 202–292.

Percequillo, A.R., Weksler, M., Costa, L.P. A new genus and species of rodent from the Brazilian Atlantic Forest (Rodentia: Cricetidae: Sigmodontinae), with comments on the Oryzomyine biogeography. *Zool J Linnean Soc.* **2011**, 161: 357-390. doi: 10.1111/j.1096-3642.2010.00643.x

Pine, R.H., Timm, R.M., Weksler, M. A newly recognized clade of trans-Andean Oryzomyini (Rodentia: Cricetidae), with description of a new genus. *J Mammal.* **2012**, 93(3): 851-870. Doi: 10.2307/23259981

Rocha, R.G., Ferreira, E., Costa, B.M.A., Martins, I.C.M., Leite, Y.L.R., Costa, L.P., Fonseca, C. Small mammals of the mid-Araguaia River in central Brazil, with the description of a new species of climbing rat. *Zootaxa*. **2011,** 2789(2789):1-34. Doi: 10.1596/9780821388358_Over

Rocha, R.G., Justino, J., Leite, Y.L.R., Costa, L.P. DNA from owl pellet bones uncovers hidden biodiversity. *Syst Biodivers*. **2015,** 13(4): 403-412. Doi: 10.1080/14772000.2015.1044048

Rocha, R.G., Duda, R., Flores, T., Rossi, R., Sampaio, I., Mendes-Oliveira, A.C., Leite, Y.L.R., Costa, L.P. Cryptic diversity in the *Oecomys roberti* complex: revalidation of *Oecomys tapajinus* (Rodentia, Cricetidae). *J Mammal*. **2018,** 99: 174–186. Doi: 10.1093/jmammal/gyx149

Rosa, C.C., Flores, T., Pieczarka, J.C., Rossi, R.V., Sampaio, I., Rissino, J.D., et al. Genetic and morphological variability in South American rodent *Oecomys* (Sigmodontinae, Rodentia): evidence for a complex of species. *J Genet*. **2012,** 91: 265–277. doi: 10.1007/s12041-012-0182-2

Saldanha, J., Ferreira, D.C., da Silva, V.F., Santos-Filho, M., Mendes-Oliveira, A.C., Rossi, R.V. Genetic diversity of *Oecomys* (Rodentia, Sigmodontinae) from the Tapajós River basin and the role of rivers as barriers for the genus in the region. *Mamm Biol.* **2019**, 97: 41–49. doi: 10.1016/j.mambio.2019.04.009

Suárez-Villota, E.Y., Carmignotto, A.P., Brandão, M.V., Percequillo, A.R., Silva, M.J.J. Systematics of the genus *Oecomys* (Sigmodontinae: Oryzomyini): molecular phylogenetic, cytogenetic and morphological approaches reveal cryptic species. *Zool J Linn Soc*. **2018,** 184(1), pp.182-210. doi: 10.1093/zoolinnean/zlx095

**Supplementary Table 2.** FISH results obtained in representatives of *Cerradomys*, *Oecomys*, *Neacomys*, *Thaptomys*, *Akodon*, *Oxymycterus* and *Blarinomys* taxa analysed with HME whole-chromosome painting probes [54].

| HME | CLA | OAU | OCA-PA | OCA-RJ | OPA-A | OPA-B | OPA-C | NVO | NEL | NXI | NMA | NPA | NSP-E | NAM | TNI | AMO | ASP | NLA | OAM | BBR |
| --- | --- | --- | --- | --- | --- | --- | --- | --- | --- | --- | --- | --- | --- | --- | --- | --- | --- | --- | --- | --- |
| 1 | 2q, 20 | 2, 19, 23q | 13, 16, 29 | 13, 17, 28 | 2, 4, 29 | 2, 4, 27 | 2, 4, 30 | 6, 8 | 2p, 4q | 6, 8 | 6, 8 | 6, 8 | 6, 8 | 6, 8 | 4, 8 | 1q dist., 4q | 1q int., 2q dist. | 5q dist., 7 | 12, 13 | 2p, 4q |
| 2 | 10, 18, 19 | 4, 22q | 4 | 1p, 4 | 9, 10 | 9, 10 | 9, 10 | 2 | 3q | 2 | 2 | 2 | 2 | 2 | 7, 12 | 1q int., 7q | 1p dist., 2q int. | 9q int., 13 | 8 | 5q |
| 3 | 1q int., 3p | 3, Xp, Y_2_ | 1 | 1q | 5, 31 | 5, 28 | 5, 31 | 3 | 2p | 3 | 3 | 3 | 3 | 3 | 1 int. and dist. | 2q | 2p dist. | 3q dist. | 1q dist. | 1q dist. |
| 4 | 5, 13 | 14, 24 | 2 | 2 | 1, 16q prox. | 1, 17q prox. | 1, 16q prox. | 1q dist. | 1q dist. | 1q dist. | 1q dist. | 1q dist. | 1q dist. | 1q dist. | 13, 15 | 1p prox., 5p dist. | 2q prox. and int., 3q int. | 1q prox., 10q prox., 11q dist. | 9, 24 | 3q |
| 5 | 1p dist., 1q prox., 8 | 7, 18, 31 | 3q dist., 15, 27 | 3q dist., 15, 27 | 15, 22, 24 | 16, 21, 23 | 15, 22, 24 | 19, 22, 24, 26p | 3p dist., 21, 22, 24 | 15q dist., 19, 24 | 15q dist., 19, 23 | 19, 26 | 9, 17 | 9, 28, 31 | 2 dist., 5 prox., 6 prox. | 3q int., 6p int., 10 | 1p int. and prox., 3q int. (ts) | 6q dist., 12q int., 14q dist. | 2p prox., 3p, 23, 25 | 7q, 9q dist. |
| 6 | 4q dist. | 21p, 30 | 9, 25, 26 | 9, 16 | 3 | 3 | 3 | 5q prox., 18 | 5q prox., 8 | 5q prox., 18 | 5q prox., 18 | 5q prox., 18 | 5q prox., 19 | 5q prox., 18 | 3 prox. and int. | 2p | 2p int. | 2q int. | 7q prox. | 2q prox. |
| 7 | 3q int. | 21q | 7 | 7 | 6 | 6 | 6 | 7, 9q int. | 3p int., 5p | 7, 9q int. | 7, 9q int. | 7, 9q int. | 7, 10q int. | 7, 10q int. | 18 | 5q prox., 8q | 1p int., 3q int. | 1q int., 4q prox. | 11q prox., 15 | 1p prox., 9p |
| 8 | 4q prox., 7 | 1 | 5 | 5 | 11 | 11 | 11 | 12, 13 | 6, 15 | 12, 13 | 12, 13 | 12, 13 | 12, 16 | 12, 16 | 6 dist. | 3p | 3q prox. | 1q int. | 10 | 4p prox., 6q |
| (9,10) | 2p dist., 3q dist. | 5, 22p, 26p | 3q prox., 12 | 3q prox., 12 | 7, 12 | 7, 12 | 7, 12 | 9q (ts), 10 | 1p, 3p int. (ts) | 9q (ts), 10 | 9q (ts), 10 | 9q (ts), 10 | 10q (ts), 14 | 10q (ts), 14 | 2 prox., 5 dist. | 5q, 9p | 1q int., 3q int. | 1q dist., 6q prox. | 11q dist., 17 | 1p dist., 8p |
| 11 | 11q prox., 6 | 9, 20 | 6q dist., 8 dist. | 6q dist.; 8q dist. | 20q dist., 28, 30, 32 | 13 | 20q dist., 27, 29 | 20, 23p | 11, 26 | 20, 22 | 20, 22q | 14, 25p | 24, 25 | 22, 23 | 9 dist., 10 prox. | 1p int., 6q dist. | 2q int., 3q dist. | 10q int., 12q dist. | 2q dist., 16q prox. | 3p dist. |
| 12 | 2p prox. | 23p | 14 | 14 | 13 | 14 | 13 | 4q prox. | 4q prox. | 4q prox. | 4q prox. | 4q prox. | 4q prox. | 4q prox. | 16 | 1q int. | 2q int. | 5q prox. | 18 | 8q |
| (13,22) | 1q (ts), 9 | 8q prox., 17, 26q | 10q dist., 18q prox., 22 | 10q dist., 19q prox., 22 | 8q dist., 14q prox. | 8q dist., 15q prox. | 8q dist., 14q prox. | 1q int., 26q, 27p | 1q int., 19, 23 | 1q int., 21, 28p | 1q int., 21, 28p | 1q int., 20, 21 | 1q int., 21, 23 | 1q int., 19, 21 | 9 prox., 11 int. and dist., 21 | 3q prox., 4p dist., 6q prox. | 1q int., 3q int. (ts) | 4q dist., 12q prox., 14q prox. | 2p dist., 3q, 14q dist. | 5p dist., 9q prox., 12 |
| 14 | 1p int., 21 | 13q dist., 15 | 3q int., 30 | 3q int., 29 | 23, 27q dist. | 22, 25q dist. | 23, 28q dist. | 23q, 25p prox. | 20, 25 | 25q, 26p prox. | 24q, 25p prox. | 16, 24p prox. | 26, 28 | 24, 26 | 17 prox., 24 | 6p prox., 8p int. | 1p int., 3q int. | 2q int., 15q int. | 21q int., 26 | 7p proximal, 13 |
| 15 | 12 | 10 | 11 | 11 | 17 | 26, 33 | 17 | 15 | 9 | 17 | 17 | 15 | 15 | 15 | 19 | 9q | 1q dist. | 8q dist. | 19 | 6p |
| (16,17) | 1q prox., 11q dist. | 6, 27 | 19, 20 | 20, 24 | 33, 34 | 31, 32 | 33, 34 | 4q dist., 16 | 4q dist., 10 | 4q dist., 16 | 4q dist., 16 | 4q dist., 25q | 4q dist., 22 | 4q dist., 20 | 10 dist., 22 | 1p dist., 3q dist. | 2p prox., 2q int., 3q int. | 2q prox., 10q dist. | 4, 16q dist. | 4p dist., 10p |
| 18 | 16 | 25q | 21 | 21 | 26 | 24 | 26 | 17 | 7 | 15q prox., | 15q prox. | 17 | 27 | 25 | 1 prox., 23 | 1q prox., 5p prox. | 2p int., 2q int., 3q int. | 3q prox., 9q prox. | 1p prox., 5 | 3p prox.,  10q |
| 19 | 1p int., 3q prox. | 12, 13q prox. | 6q int., 28 | 6q int., 26 | 16q dist., 27q prox. | 17q dist., 25q prox. | 16q dist., 28q prox. | 14, 25p dist. | 18 | 14, 26p dist. | 14, 25p dist. | 24p dist., 24q | 18 | 17 | 17 dist. | 8p dist., 8q prox. | 1p int. (ts) | 15q prox. and dist. | 21q (prox. and dist.) | 7p dist. |
| 20 | 1q dist. | 25p prox. | 10q prox. | 10q prox. | 8q prox., 20q prox. | 8q prox. | 8q prox., 20q prox. | 1q prox. | 1q prox. | 1q prox. | 1q prox. | 1q prox. | 1q prox. | 1q prox. | 11 prox. | 4q prox. | 1q prox. and int. | 4q int. | 14q prox. | 5p prox. |
| 21 | 4p, 4q int. | 8q dist. | 18q dist. | 19q dist. | 14q dist. | 15q dist. | 14q dist. | 5q dist. | 5q dist. | 5q dist. | 5q dist. | 5q dist. | 5q dist. | 5q dist. | 3 dist. | 2p dist. | 2p int. | 2q dist. | 7q dist. | 2q dist. |
| 23 | 15 | 16, 29 | 6q prox., 23 | 6q prox., 23 | 19, 21 | 19, 20 | 19, 21 | 21, 25q | 14, 16 | 23, 26q | 25q, 26q | 23 | 13, 20 | 13, 29 | 20 | 7p | 1p int. | 8q prox. | 20 | 3p int. |
| 24 | 14 | 11 | 17 | 18 | 18 | 18 | 18 | 11 | 12 | 11 | 11 | 11 | 11 | 11 | 14 | 6p dist. | 3q int. (ts) | 9q dist. | 22 | 11q |
| 25 | 17 | 28 | 24 | 25 | 25 | 29, 30 | 25 | 28 | 13 | 27 | 27 | 27 | 29 | 27 | 1 prox. | 2p prox. | 2p int., 3q int. | 3q int., 11q prox. | 1p dist., 1q int. | 1q prox. |
| 26 | 22 | 25p dist. | 8q prox. | 8q prox. | 35 (h) | 34 (h) | 32 | 27q | 17 | 28q | 28q | 22 | 30 | 30 | 25 | 11 | 4 | 16 | 6 | 11p |
| X | X | Xq | Xq | Xq | Xq | Xq | Xq | Xq | X | Xq | Xq | X | Xq | Xq | X | X (Xq) | X | X | Xq | X |

Short arm (p). Long arm (q). Proximal (prox). Interstitial (int). Distal (dist). Two segments (ts). Heteromorphic pair (h). *Hylaeamys megacephalus* (HME), *Cerradomys langguthi* (CLA) [54], *Oecomys auyantepui* (OAU) (present study), *O. catherinae-*Pará (OCA-PA), *O. catherinae-*Rio de Janeiro (OCA-RJ) [47], *Oecomys paricola* cytotype A (OPA-A), *Oecomys paricola* cytotype B (OPA-B), *Oecomys paricola* cytotype C (OPA-C) [50], *Neacomys* *vossi* (NVO), *Neacomys* *elieceri* (NEL) [57]), *Neacomys* *xingu* (NXI), *Neacomys* *marajoara* (NMA), *N. paracou* (NPA), *Neacomys* sp. E (NSP-E), *N. amoenus* (NAM) [58], *Thaptomys nigrita* (TNI), *Akodon montensis* (AMO) [55], *Akodon* sp. (ASP), *Necromys lasiurus* (NLA) [56], *Oxymycterus* *amazonicus* (OAM), *Blarinomys* *breviceps* (BBR) [59].
